# A novel life cycle assessment and life cycle costing framework for carbon fibre-reinforced composite materials in the aviation industry

**DOI:** 10.1007/s11367-023-02164-y

**Published:** 2023-03-28

**Authors:** Minghui Wu, Jhuma Sadhukhan, Richard Murphy, Ujjwal Bharadwaj, Xiaofei Cui

**Affiliations:** 1 Centre for Environment and Sustainability, University of Surrey, Guildford, UK; 2 Asset Integrity Management Section, TWI Ltd, Granta Park, Cambridge, UK

**Keywords:** Life cycle costing, Life cycle assessment, Recycling, Composite material, Aircraft door, Eco-efficiency analysis

## Abstract

**Purpose:**

Carbon fibre-reinforced composite materials offer superior mechanical properties and lower weight than conventional metal products. However, relatively, little is known about the environmental impacts and economic costs associated with composite products displacing conventional metal products. The purpose of this study is to develop an integrated life cycle assessment and life cycle costing framework for composite materials in the aviation industry.

**Methods:**

An integrated life cycle assessment (LCA) and life cycle costing (LCC) framework has been developed. The displacement of a conventional aluminium door for an aircraft by a composite door is presented as an example of the use of this framework. A graphical visualisation tool is proposed to model the integrated environmental and economic performances of this displacement. LCA and LCC models for composite applications are developed accordingly. The environmental hotspots are identified, and the sensitivity of the environmental impact results to the different composite waste treatment routes is performed. Subsequently, the research suggests a learning curve to analyse the unit price for competitive mass production. Sensitivity analysis and Monte Carlo simulation have been applied to demonstrate the cost result changes caused by data uncertainty.

**Results:**

Energy consumption was the hotspot, and the choice of composite waste treatment routes had a negligible effect on the LCA outcomes. Concerning the costs, the most significant cost contribution for the unit door production was labour. The future door production cost was decreased by about 29% based on the learning curve theory. The uncertainties associated with the variables could lead to variations in the production cost of up to about 16%. The comparison between the two doors shows that the composite door had higher potential environmental impacts and cost compared to the conventional aluminium door during the production stage. However, the composite door would have better environmental and financial performance if a weight reduction of 47% was achieved in future designs.

**Conclusions:**

The proposed framework and relevant analysis models were applied through a case study in the aerospace industry, creating a site-specific database for the community to support material selection and product development. The graphical tool was proved to be useful in representing a graphical visualisation comparison based on the integration of the LCA and LCC results of potential modifications to the composite door against the reference door, providing understandable information to the decision-makers.

**Supplementary Information:**

The online version contains supplementary material available at 10.1007/s11367-023-02164-y.

## Introduction

Composite materials have been widely used in the aeronautical industry over the last decade due to their favourable mechanical properties and low weight (Fera et al. 2020). Carbon fibre-reinforced thermoset composites dominate the market, accounting for more than two-thirds of the aeronautical industry (Cousins et al. 2019). However, there are some concerns regarding thermoset composites, such as their curing cycles leading to low production rates and end-of-life issues due to limited recyclability (Katsiropoulos and Pantelakis 2020). Therefore, the use of carbon fibre thermoplastics composite has been growing more rapidly in recent years (Nishida et al. 2018).

Life cycle assessment (LCA) and life cycle costing (LCC) are the most valuable tools for the assessment of environmental and cost performance, respectively, to enable stakeholders to improve the selection of material and production processes for product development (Witik et al. 2012). LCA is best known for quantitative analysis of the potential environmental impacts of a product or service throughout its entire life cycle. It is an internationally standardised method under ISO 14040 series (International Organization for Standardization 2006a, b). Focusing on the aviation sector, stand-alone LCA has been widely used to assess environmental impacts associated with life cycle stages (Timmis et al. 2015; Vieira and Bravo 2016). However, some limitations in the recent research, such as single impact category analysis and the rarely communicated data uncertainty, lead to a lack of confidence in the result. LCC is a method of economic analysis for all costs related to a product or service throughout its entire life cycle. Traditionally, it is based on purely economic evaluation, considering various costs associated with a product that is born directly by a given actor and usually determined by the perspective of a single actor (David et al. 2008). Therefore, costs have to be calculated in an actor-specific way. However, a methodology for cost analysis, named environmental LCC, had been developed by the Society of Environmental Toxicology and Chemistry (SETAC), which is applied to be compatible with LCA (David et al. 2008; Swarr et al. 2011). It aims to provide a methodology to merge traditional LCC with LCA efficiently and consistently. It is an extension of the conventional LCC concept, which is seen alongside LCA as two of the three main pillars in evaluating sustainability. The environmental LCC considers all costs associated with the life cycle of the product/system directly covered by one or more of the players involved in the supply chains (i.e. supplier, producer, user, customer or final disposer) (Barke et al. 2020). In our research, the LCC refers to environmental LCC. The criteria in economic spheres have shown a wide variation concerning stakeholder interests (Barke et al. 2020). In the aviation sector, this is of interest when making acquisition decisions, but aircraft producers are willing to use LCC to assess their product’s design and manufacture (Zhao et al. 2015; Hueber et al. 2016). Despite this, few published studies have focused on the development of cost models.

There has also been a growing interest in the need to consider environmental and economic dimensions together to make the best possible decisions (Zanghelini et al. 2018). For the aircraft sector, very little has been reported on applying integrated LCA and LCC for combining different life cycle aspects. A most recent study by La Rosa et al. (2021) conducted LCA and LCC of the chemical recycling of a specific type of carbon fibre-reinforced thermoset composite panel at a laboratory scale. The study featured a sensitivity analysis, investigating the possibility of change in the yield of the recovered by-product’s epoxy-thermoplastic. Detailed LCA and LCC studies were conducted, but no explicit integration was performed as part of this study. Markatos and Pantelakis (2022) developed an indexing tool to assess and rank two hypothetical aluminium and CFRP components by considering their economic, environmental and property aspects via a total score by using the analytical hierarchy process (AHP) method (Markatos and Pantelakis 2022). They pointed out that the accuracy of the output values was influenced by assumptions and data reliability. Unfortunately, only the aggregated single score for the components’ economic and environmental impacts was given; thus, it lacks in transparency, providing limited information to support decision-making.

Across all types of integration of environmental, economic and other indicators, multiple-criteria decision analysis (MCDA) and eco-efficiency methods are the most popular (Miah et al. 2017). In terms of MCDA, it was found that the most widely used MCDA methods were the weighted sum model (WSM), technique for order of preference by similarity to ideal solution (TOPSIS) and the analytical hierarchy process (AHP) (Petrillo et al. 2016; Zanghelini et al. 2018; Ling et al. 2021). It was found that the major drawbacks of combining LCA and LCC via MCDA are the subjective evaluation of criteria (Bierer et al. 2015) (i.e. determining the weights of different criteria by preference) and potential inconsistencies between judgement and ranking criteria (Miah et al. 2017). Eco-efficiency (EE) analysis (Heijungs 2022) is another standard method to compare the techniques or choice of alternatives by integrating LCC and LCA. This method has been demonstrated in many sectors, such as chemical sectors (Mangili and Prata 2020), household goods (Rüdenauer et al. 2005) and solid waste management (Abrate et al. 2014). Usually, EE is classically represented by a two-dimensional graph. The environmental and economic impacts are set on the *x* and *y* axes, respectively, and assessment results for the alternatives and reference are located in the graph. Therefore, diversity EE indexes and different quadrants are defined to represent the comparison results. However, some drawbacks exist in these studies. For example, it is more difficult to interpret the results due to the complex or unclear definitions of dividing lines/quadrants. Therefore, further research is required to explore the different EE indexes or graphical tools to present the results. Apart from MCDA and eco-efficiency, some studies have considered integrating LCA and LCC as part of an overarching framework (Kendall et al. 2008; Hong et al. 2014). However, the main limitation was that the presented results only focused on one dimension of LCA or LCC, not effectively supporting decision-making. Overall, the literature on integrating LCA and LCC reveals no consistent, preferred methodological approach to integrate LCA and LCC. The integrated LCA and LCC methods demonstrated are developed to meet their research and project needs and still need to explore (Zanghelini et al. 2018).

This paper first presents an integrated LCA and LCC modelling framework to quantify the environmental and economic impacts of a newly developed composite aircraft door for ATR 72, a turboprop-powered regional airliner. The application of the integrated LCA and LCC modelling framework to such a newly developed composite door is for the first time in the academic literature. A graphical visualisation tool that combines environmental and economic footprints evaluates current performance and improvements, applied to both door systems. Meanwhile, the EE index is proposed to rank the different alternatives. In addition, LCA and LCC models are developed to assess the environmental and economic performances of aircraft structures. Furthermore, a cost analysis on the door mass production conducted using the learning curve approach is presented. An endpoint of the learning period is defined, and a method to determine this endpoint is also developed. Sensitivity and uncertainty analyses are carried out to demonstrate the resulting changes caused by data uncertainty.

## Methods

The materials and methods for this LCA and LCC research and its framework development are presented in two parts. First, the underpinning background and methodological framework are presented, followed by the description of a graphical visualisation tool: EE analysis.

### Modelling the framework for quantifying the products’ potential environmental and economic impacts

#### Rationale for using the EE approach in the proposed framework

To the best of the authors’ knowledge, there exists a lack of a framework in the aviation industry capable of integrating LCA and LCC. An extensive literature review on the integration of LCA and LCC used in other sectors highlights their features and limitations. The key features of the framework for the integration of LCA and LCC in the aviation sector for this research include the following:

(I) Consistent system boundaries and the framework aligned with ISO 14044
(II) Evaluation of both environment and economic sides in detail by process-based models
(III) The time dimension of cost values
(IV) Evaluation of wider impact categories used in LCA
(V) Data transparency evaluations by sensitivity and uncertainty analysis
(VI) Integration of LCA and LCC via EE analysis
(VII) Visualising the performance of alternatives and improvements by mapping the environment and economic efficiency in two dimensions

#### Characteristics of the framework for modelling LCA and LCC

The proposed approach is based on a process viewpoint to investigate the LCA and LCC of a product at process levels across the whole life cycle. ‘Process’ describes the various activities from the extraction of raw materials, material-working processes, inspection of semi-finished parts, the assembly of semi-finished parts to make a final product, in-service repair, use and maintenance and recycling or final disposal.

**Fig. 1:**
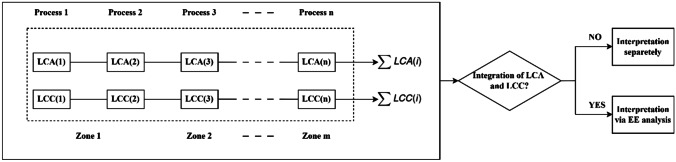
An integrated framework combining environmental LCA and LCC

In this approach, a common basis for sharing the same set of system boundaries and data sources is established for the inventory for the LCA and LCC is used to reduce double work in the data acquisition and minimise data inconsistency limiting the significance of the results. As depicted in Fig. 1, the environmental and economic impacts caused by various processes within a product are categorised into environmental and economic assessments. The LCA and LCC refer to the assessment of environmental and economic impacts, respectively. The total environmental and economic impacts are the sum of all the environmental burdens in the processes and all the costs caused by the processes, respectively. In addition, a whole life cycle can also be considered several ‘zones’ where a ‘zone’ is defined as a life cycle stage within a product. A zone may include several processes and/or a single process. For example, a product is defined as having zones of production, use and end-of-life treatment stages.

This simulation model is further required to confirm whether the preference of different criteria (LCC and LCA) needs to be considered.

A parallel presentation of results is performed in the evaluation and interpretation step if the answer is no. Normally, the key findings for LCA and LCC can be interpreted independently to highlight the environmental and economic hotspots within specific life cycle stages and, overall, for the whole life cycle. Such hotspot processes can then be examined to model options to improve their efficiency or, where possible, their replacement with alternatives that have less impact on the life cycle.

If the answer to the integration step in the framework is yes, then a system integration is performed by eco-efficiency (EE) analysis. It is usually used to determine the ‘best’ alternative from a group of alternatives. A joint evaluation of the calculated economic and environmental results is required. A picture of both the environmental and economic sides of products and processes is given via results presentation in a two-dimensional graph. A graphic tool is proposed to evaluate current performance and any improvements in this research; see Sect. 2.2 for further details.

Both LCA and LCC are data-intensive techniques that require detailed information about the analysed product, but an absence or limited access to some data might exist during the assessments. Conversely, a product concept is ever-changing due to the development of technology or legislation changes regarding the disposal of the product. Therefore, consideration of variability and uncertainty in the input data by uncertainty and sensitivity analysis is needed to support a transparent assessment. Options to address this include local sensitivity analysis, global sensitivity analysis, scenario analysis and probabilistic approaches.

This integrated approach seeks to provide researchers and decision-makers with a transparent basis to explore the factors causing environmental and cost impacts and target recommendations for improvements to the life cycle of the analysed product. Such improvements can include an opportunity for further eco-design, selection of materials, planning and management of processing. In the following section, this illustrated framework is applied to a real composite aircraft door case study, focusing on the manufacturing stage within the life cycle.

### A graphical visualisation tool: EE analysis

The EE performance coordinate plane is introduced as a decision-making support tool to help with material technology/system selection as well as to support comparison/prioritisation of the alternative scenarios that improve the processes. Some examples of past works show combined economic and environmental marginal analysis at product levels in a complex network (Martinez-Hernandez et al. 2014). However, the present work is distinctive because of its EE performance considerations to compare alternative products. It plots the comparison of the scenarios on their environmental and economic performance or ‘eco-efficiency’. In addition, one drawback of the traditional eco-efficiency graphs is the difficulties of the interpretation, as mentioned previously. It provides a quick tool to analyse the alternatives for its economic and environmental performance improvements against a reference.

The results from LCA and LCC are used as a basis to assess the environmental benefits in parallel with the economic benefits. The reference point (e.g. traditional system) as the origin (0, 0) is located at the centre of the coordinate plane. Point *P* is the analysed system represented by a pair of numbers (*x*_*a*_, *y*_*a*_), whose values are calculated in Eqs. 1 and 2:

1 $${x}_{a}=\frac{{E}_{r}{-E}_{a}}{{E}_{r}}$$

2 $${y}_{a}=\frac{{EI}_{r}{-EI}_{a}}{{EI}_{r}}$$

where *E*_*a*_ is the economic performance for the alternative product/system; *E*_*r*_ is the economic performance for the reference product/system; *EI*_*a*_ is the environmental performance for the alternative product/system; and *EI*_*r*_ is the environmental performance for the reference product/system.

Therefore, the *x*-axis represents economic benefits as a change in economic performance by shifting the reference product/system to an alternative product/system. Similarly, the *y*-axis represents the environment benefits as a change in the system’s environmental performance by shifting the reference product/system to an alternative product/system. It is worth noting that the ‘benefits’ can be a negative value, which represents a worse performance by replacing the traditional product/system. This expression holds the common meaning of performance, that is to say, the higher number is better.

The diagram presents four quadrants, where each quadrant can be characterised as:

(I) Quadrant I (+ , +): the alternative product/system is better than the reference one in terms of both environmental and economic performance. In this quadrant, the alternative provides a positive EE solution.
(II) Quadrant II (− , +): the alternative product/system performs better in terms of environmental performance but costs more than the reference. Results in this quadrant indicate improvement actions should be undertaken to increase the economic performance if considering a shift from the reference to the alternative.
(III) Quadrant III (− , −): the alternative product/system is worse than the reference in both environmental and economic performance. Results in this quadrant indicate that a shift from reference to alternative would be a worse EE solution, and improvement actions are highly recommended for both environmental and economic parameters if a shift is to be contemplated.
(IV) Quadrant IV (+ , −): the alternative product/system is worse in environmental performance but costs less than the reference. Results in this quadrant indicate improvement actions are needed to migrate the environmental emissions if shifting from the reference to the alternative.

The graphic depiction and the numerical scoring for EE enable analysts to discuss both the direction and the extent of the advantages and disadvantages of alternatives for changing the environment and economic performance in a specific and comprehensive way.

First, the LCA and LCC performances of the functional unit for alternative materials were examined. Second, the comparison analysis was performed, and the EE graph was plotted.

To further evaluate alternatives, the following equation for EE index can be calculated using Eq. 3:

3 $$EE=A{x}_{i}+B{y}_{i}, \quad i=\mathrm{1,2},\cdots ,m$$

where *A* is weight of economic performance, *B* is weight factor of environmental performance, and *A* and *B* satisfy Eq. 4:

4 $$A+B=1$$

The weight factors are subjectively quantified and should be determined by decision-makers’ perspectives. Then all alternatives are ranked, and the best alternative can be found with the highest EE score.

## Case study

### Goal and scope

This case study aims to quantify and evaluate the economic and environmental performances of the production of the composite door and identify the critical processes and materials with the highest environmental and economic burdens. Moreover, the economic and environmental impacts of the composite door are compared to its metallic counterparts to assess the potential benefits or limitations.

The functional unit is defined as manufacturing one door for the ATR 72 aircraft.

**Fig. 2:**
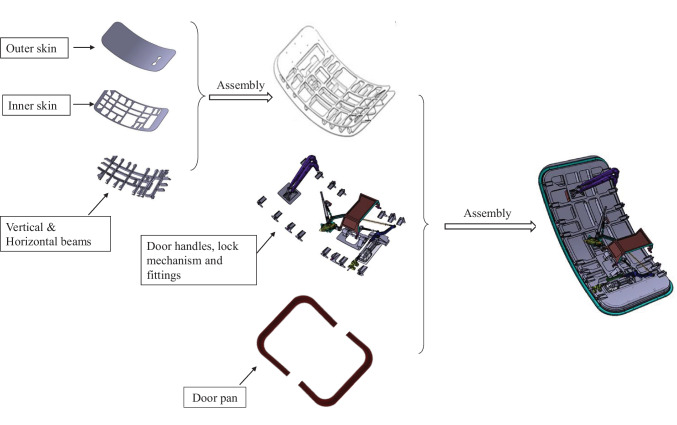
The schematic diagram of the composite door

The composite door consists of the outer skin, inner skin beams, door pan and lock mechanism and fittings; see Fig. 2. They are made of different materials: carbon fibre-reinforced thermoplastic (TP), carbon fibre-reinforced thermoset (TS), aluminium alloy (Al) and 18/8 stainless steel (SS). The aluminium door mainly consists of two parts: the body and the fittings, i.e. handles and lock mechanism. The main difference between the aluminium and composite doors is the material and the production processes used in the door body part production, while that of fittings in these two doors are the same but different amounts of raw materials used in the door production. The detailed raw materials and each manufacturing process are described in Appendix Table i.

System boundaries defined in this work focus primarily on the production process (cradle-to-gate). This cradle-to-gate system is extended further by the modelling of potential end-of-life composite management processes (see Sect. 3.2.1). Scraps considered in this model are defined as those generated during the production process. They can be categorised into either co-product or waste. The economic allocation method was used to allocate the impacts on the waste or co-products: those scraps that can be sold or reused and that benefit the manufacturer can be considered as co-products. In comparison, waste is defined as those that the manufacturer cannot treat and will cause additional costs to involve a dealer for further disposal. The dealer will select the proper disposal technique, such as recycling and landfill, based on the economic and environmental issues.

This study treats all TP and TS scraps generated from the production processes as waste and sent to the end-of-life composite waste management. However, the specific composites disposal or recycling routes are unknown because data could not be obtained. In addition, no specific data on waste recycling/incineration of carbon fibre and resin have been identified (Schmidt et al. 2014). Therefore, based on the literature, TP and TS scraps are assumed to be recycled via mechanical recycling (Hadigheh et al. 2021). Also, it is assumed that recycled fibres can replace the same volume of virgin glass fibre as fillers or reinforcement in lower-quality applications (Witik et al. 2013), thus avoiding the energy and environmental impacts of virgin glass fibre production. This high recovery efficiency value maximises the recycling substitution credits in the analysis. The other composite waste treatment methods, such as pyrolysis recycling, solvolysis recycling, incineration and landfill, were also tested as a sensitivity analysis in Sect. 4.4.1.

Al and SS scraps are treated as co-products because they have residual values and are assumed to leave the system burden free to another product life cycle. Al and SS components have a fairly high ‘buy-to-fly’ ratio, i.e. high-volume scraps generated during production due to the complexity of the structural parts production using CNC (MIT Publishing 2010); larger scrap volumes would be expected for more complex components. We are aware that there is some literature (Timmis et al. 2014; Lockett 2019) where this ratio can reach 25:1 (20:1 in the present study for Al and SS from primary data). Of course, metal scraps can be recycled but have low value and cannot be used in aircraft applications (Das and Gilbert Kaufman 2008; Woidasky et al. 2017). Therefore, the metal scraps are represented here as relatively high-volume but low-value co-products.

The manufacturing processes are developed based on today’s advanced and emerging technology, and it is still unknown if they will be changed in the future. However, the present study assumes no changes in these processes for future mass production.

### Life cycle assessment

#### Inventory analysis and sources

The process data for LCA and LCC are collected simultaneously to minimise double work in the data acquisition and avoid data inconsistency. In this section, only the LCA related data will be discussed (LCC is discussed in Sect. 3.3).

Both primary and secondary life cycle inventory (LCI) data are used in the LCA analysis. The primary data obtained from the manufacturers contain the amount of produced and consumed materials and the direct electricity used at the production site. Experienced experts have validated these data. The secondary data is extracted from some latest literature and ecoinvent 3.8 compiled in November 2021 under SimaPro 9.3. For those data which are not available in ecoinvent 3.8, the European Life Cycle Database (ELCD) (European Commission 2018), developed by the Joint Research Centre of the European Commission, is used.

Publicly available LCI data on virgin carbon fibre production is sparse (Vita et al. 2019). In particular, the data on the production of polyacrylonitrile (PAN), the precursor, is based on the ecoinvent 3.8 database. For carbon fibre manufacture, Khalil (Khalil 2017) detailed the production processes and data collection of PAN fibre oxidative stabilisation and carbonisation; this data was adopted for the present study. The prepregs have been modelled based on material specifications stated in Sect. 3.1. The electric energy consumption of the prepregs manufacturing was obtained from Song et al. (2009). The remaining inventory data for door production, including the quantities of materials needed, the scraps generated, labour hours and electric energy consumption in each production activity, are based on industry data obtained from project manufacturers. The material and process datasets used in the SimaPro 9.3 model selected for composite door manufacturing are shown in Appendix Table ii. The material and energy flows are calculated and depicted in Appendix Fig. i.

In terms of allocation procedures for the manufacture of both Al and SS components, economic allocation (Li et al. 2019) is used to allocate the environmental burdens. That means part of the burdens are attributed to the main product, and the remaining is allocated to the co-product according to their economic ratio. The allocation data are given in Table 1.

**Table 1:** Allocation percentage used for Al components’ manufacturing and SS components’ manufacturing

**Product**	**Mass produced**	**Price**	**Allocation by economic value**
Al components	15 kg	30.3 €/kg	73%
Al scraps	285 kg	0.6 €/kg	27%
SS components	5 kg	13.6 €/kg	64%
SS scraps	95 kg	0.4 €/kg	36%

In terms of its conventional counterparts, the inventory data were provided by the door manufacturer. The material and energy flow with respect to the door production processes are depicted in Appendix Fig. ii. The material and process datasets used in the SimaPro 9.3 model selected for aluminium door manufacturing are shown in Appendix Table iii. The scraps were generated from cutting, machining and stamping processes; the buy-to-fly ratio was reduced to 2:1 for aluminium door body production, according to the manufacturer. Therefore, the allocation of Al components and Al scraps of the door body is 98% and 2%, respectively. The economic allocation ratio on the Al and SS components in the conventional door is the same as that of the composite door.

The transport distances of doors’ manufacturing and the LCI data for energy use are specific. They were determined based on the actual sites from which the feedstocks were sourced and where the aircraft doors’ manufacturing was applied. The distances were calculated using route planners and map services, e.g. Google Earth. And the average country grid mix from ecoinvent 3.8 was selected for energy data.

#### Life cycle impact assessment

The LCA software SimaPro 9.3 assimilates resources and emissions data for the datasets shown in Appendix Tables ii and iii. The life cycle impact assessment was performed using the Impact 2002+ methodology (Jolliet et al. 2003). In addition to a wide range of individual atmospheric, land and aquatic life cycle impact characterisations, the Impact 2002+ methodology groups the individual impact characterisations into four damage categories. The total environmental impact is estimated by normalising and weighting the results to a single score in unit points (Pt), i.e. Pt is equal to ‘pers·yr’. One Pt represents the average impact in a specific category caused by one European for 1 year (Scelsi et al. 2011).

### Life cycle costing

LCC and LCA share the same system boundary.

#### Cost modelling

The cost involves material, labour, energy, waste treatment, equipment, tooling and transportation. These cost elements should be considered for each operation included in the production process. The summation of each element for all the production processes gives the total cost required for each activity performed. The cost for each operation is summed to get the total cost using Eq. 5:

5 $$\begin{aligned}Production\; Cost=&\;\textstyle\sum Material\; Cost+\textstyle\sum Labour \;Cost\\&+\textstyle\sum Energy\; Cost+\textstyle\sum Scrap \;treatment\;Cost\\&+\textstyle\sum Equipment\; Cost+\textstyle\sum Tooling\; Cost\\&+\textstyle\sum Transport \;Cost\end{aligned}$$

The methods to estimate each cost element in Eq. 5 are detailed in the following section.

##### Material cost

The material used for composite door manufacturing consists of the raw materials in each operation and consumables during production, mainly including vacuum bagging materials, breather fabric, release agents and solvent. However, compared to material expenses, consumables cost less and are challenging to be recorded in the aerospace industry. Thus, a typical ratio of 3% is applied to quantify the relation between the consumables’ cost and the raw material cost (Ma 2011).

The calculation of raw material cost should consider the scrap rate and the reject rate. The raw material cost, ${C}_{RawM}$, can be calculated using Eq. 6:

6 $${C}_{RawM}=\frac{{W}_{C}}{\left(1-{R}_{scrap}\right)\left(1-{R}_{reject}\right)}\times {U}_{M}$$

where ${W}_{C}$, ${R}_{scrap}$, ${R}_{reject}$ and ${U}_{M}$ stand for the weight of the formed part, the material scrap rate, the part reject rate and the material unit price, respectively.

The consumable cost, ${C}_{Cons.}$, can be calculated as in Eq. 7:

7 $${C}_{Cons.}=3\% \;{\times \;C}_{RawM}$$

##### Labour cost

The labour cost is categorised as direct labour cost and indirect labour cost.

The direct labour cost for a certain process, ${C}_{LD}$, for a specific process can be estimated using Eq. 8:

8 $${C}_{LD}={R}_{L}\times {T}_{L}\times {N}_{Oper.}$$

where ${R}_{L}$, ${T}_{L}$ and ${N}_{Oper.}$ stand for the labour rate, the labour hours and the number of operators required, respectively.

Alternatively, the lay-up hours for manual lay-up of prepregs can be approximated (Hagnell and Åkermo 2015) (Eq. 9):

9 $${T}_{L}=\frac{W}{{R}_{Lay}}$$

where ${R}_{Lay}$ stands for lay-up rate.

The indirect labour cost is the cost that is not directly related to the production activity and the performance of services. It represents the labour supervision and labour fringe benefits needed to support the level of operations. The indirect labour cost is difficult to be determined and varies in different companies. Generally, such costs are distributed over the various operations based on direct labour costs (Bernet et al. 2002). In this model, the ratio of indirect labour cost, ${ C}_{LI}$, to the direct labour cost in the aerospace industry, is assumed to be 48% (Bernet et al. 2002; Ma 2011) (Eq. 10):

10 $${C}_{LI}=48\mathrm{\% }\times { C}_{LD}$$

##### Energy cost

The electricity cost, ${C}_{E}$, is estimated using Eq. 11:

11 $${C}_{E}=P\times {T}_{M}\times {U}_{EP}$$

where ${U}_{EP}$, $P$ and ${T}_{M}$ stand for the electricity unit price, the equipment operation power and machine operating time, respectively.

Alternatively, ${C}_{E}$ can also be estimated using the energy intensity (Eq. 12):

12 $${C}_{E}=\rho \times W\times {U}_{EP}$$

where *ρ* and $W$ stand for the energy intensity and the weight of component or material, respectively.

##### Scrap treatment cost

As mentioned previously, scraps can be categorised into either co-product or waste.

Scrap treatment cost for treating co-product ${C}_{ST}$ can be estimated using Eq. 13:

13 $${C}_{ST}={W}_{S}\times {U}_{ST}$$

where ${W}_{S}$ and ${U}_{ST}$ stand for the weight of the scraps and the unit cost of scraps treatment, respectively.

The waste treatment (i.e. end-of-life waste management in this research) of aircraft products mainly includes three processes: (i) demolition, (ii) transportation to a waste treatment site and (iii) waste treatment process (i.e. recycling, landfill or incineration). The waste treatment cost also includes selling the recyclates to make revenues. Therefore, for each waste treatment route, the cost is determined as the summation of all the costs, including demolition, transport, waste treatment process and revenues.

Therefore, scrap treatment cost for treating waste ${C}_{WT}$ can be estimated using Eq. 14:

14 $${C}_{WT}={W}_{W}\times {(U}_{WT}-{U}_{rS})$$

where ${W}_{W}$, *U*_*WT*_ and *U*_*rS*_ stand for the weight of the treated waste, the unit cost per mass unit of waste treatment and the unit cost per mass unit of recovered substance, i.e. energy or material, respectively. It should be noted that the unit cost of each waste treatment technique is an average unit cost which should consider the capital cost and the cost of details of activities required by each waste treatment technique, such as labour, energy, equipment and transport costs.

##### Equipment cost

The equipment cost is the investment costs for machines used in the manufacturing components of the door product. Based on life cycle cost theory, each piece of equipment should consider the installation cost, training cost, purchased cost, maintenance cost (including the cost of labour, materials, repairs and scheduled maintenance), utility cost during the operation (such as electricity, water coolant) and salvage value (Eq. 15):

15 $${C}_{EL}={{C}_{Euip.IT}+C}_{Euip.P}+{C}_{Euip.M}+{C}_{Euip.O}-{C}_{Euip.S}$$

where ${C}_{Euip.P}$ is the purchased cost of the equipment, ${C}_{Euip.IT}$ is the installation and training cost to purchased cost, ${C}_{Euip.M}$ is the machine maintenance cost, ${C}_{Euip.O}$ is the operation cost, and ${C}_{Euip.S}$ is the salvage value.

Generally speaking, the installation and training costs are usually expressed as a percentage of machine price (Haffner 2002). The cost for machine maintenance is estimated based on surveying manufacturers/equipment suppliers or consulting industry experts to get relative costs (Thomas 2018). It also can be a percentage of the annual capital cost for the company or the project or a function of parts production volume (Haffner 2002). The machine operation cost is mainly the energy cost when producing the parts based on energy consumption. Salvage value or residual value is estimated as a worth value at the end of its useful life.

It is difficult for an estimator to judge the exact hours for which the equipment will be used in a day for a specific part. Therefore, it is hard to estimate the total cost of equipment as the previous component cost model by distributing the total number of the parts performed within a given lifetime of the equipment. In this case, estimating equipment cost for a part by considering the hourly cost of the different equipment is feasible.

Therefore, allocating the machine cost to each component of the product is solved here by introducing an hourly machine rate as defined in Eq. 16:

16 $${R}_{Machine}=\frac{{C}_{EL}}{{T}_{Oper.T}}$$

where ${R}_{Machine}$, ${C}_{EL}$ and ${T}_{Oper.T}$ stand for the hourly machine rate, the life cycle cost of equipment and the expected total hours of operating life, respectively. Therefore, the equipment cost for each component or part is calculated by multiplying the running hours of the machine by the machine hour rate, shown in Eq. 17:

17 $${C}_{Euip.}={R}_{Machine} \times T$$

where ${C}_{Euip.}$ and $T$ stand for the equipment cost for each component and the operating time, respectively.

##### Tooling cost

The tooling cost estimation is similar to that of equipment cost. Tooling refers to the tools or moulds used to produce the components of the product. It is always designed specifically for individual component production, and the cost varies depending on quantities, material type, finished part specification and the quality of tools produced (Lauzier 2022). The accurate estimation for tooling entirely relies on stakeholder data collection surveys. It should be handled in the same way as the equipment cost considering purchased/production cost, maintenance cost, operation cost and salvage value through the useful life of the tooling. Distributing the tooling cost to each component is defined by introducing a tooling rate, as stated in Eq. 18:

18 $${R}_{Tooling}=\frac{{C}_{ToolingL}}{{T}_{Service.T}}$$

where ${R}_{Tooling}$, ${C}_{ToolingL}$ and ${T}_{Service.T}$ are the tooling hourly rate, the life cycle cost of the tooling and the expected total hours of service life, respectively.

Therefore, the tooling cost for each component or part, ${C}_{Tooling}$, is estimated using Eq. 19:

19 $${C}_{Tooling}={R}_{Tooling} \times T$$

##### Transport cost

The transport cost, ${C}_{T}$, is estimated using Eq. 20.

20 $${C}_{T}=W\times d\times {U}_{T}$$

where *W*, *d* and ${U}_{T}$ stand for the weight of the load, the travel distance and the unit price for the transport, respectively. The unit price for transporting the load is abbreviated as €/t-km. Here, t-km, i.e. a tonne-kilometre, is a unit of measure of freight transport which represents the transport of one tonne of goods by a given transport mode (road, rail, air, sea etc.) over a distance of 1 km.

#### Multiple production lots

##### Learning curve and slope

During the production period for a new product, the labour hours required for unit production are generally expected to decrease as the cumulative production quantity increases due to the improved proficiency of a workman with practice (Benkard 2000; Zhou et al. 2019). The relationship between the labour hours for unit production and the production quantity can be quantified by a product improvement curve, commonly referred to as a learning curve (Krajewski et al. 2010), following a power function (Eq. 21):

21 $$y=a \;{x}^{\mathrm{b}}$$

where $x$ is the cumulative number of units produced. $y$ is the labour hours (or cost) required to produce the *i*^*th*^ unit. $a$ is the labour hours (or cost) required to produce the first unit ($x=1$). $b$ is the learning coefficient measuring the rate at which the labour hours (or cost) are reduced as cumulative output increases.

The slope of the learning curve, *v*, which represents the amount (fraction) that costs decrease as the cumulative production quantity doubles, is estimated (Eq. 22):

22 $$v={2}^{b}$$

If *b* = 0, the labour hours required to produce a unit are constant, being at $a$, which means no learning from previous experience.

##### Learning and standard period

**Fig. 3:**
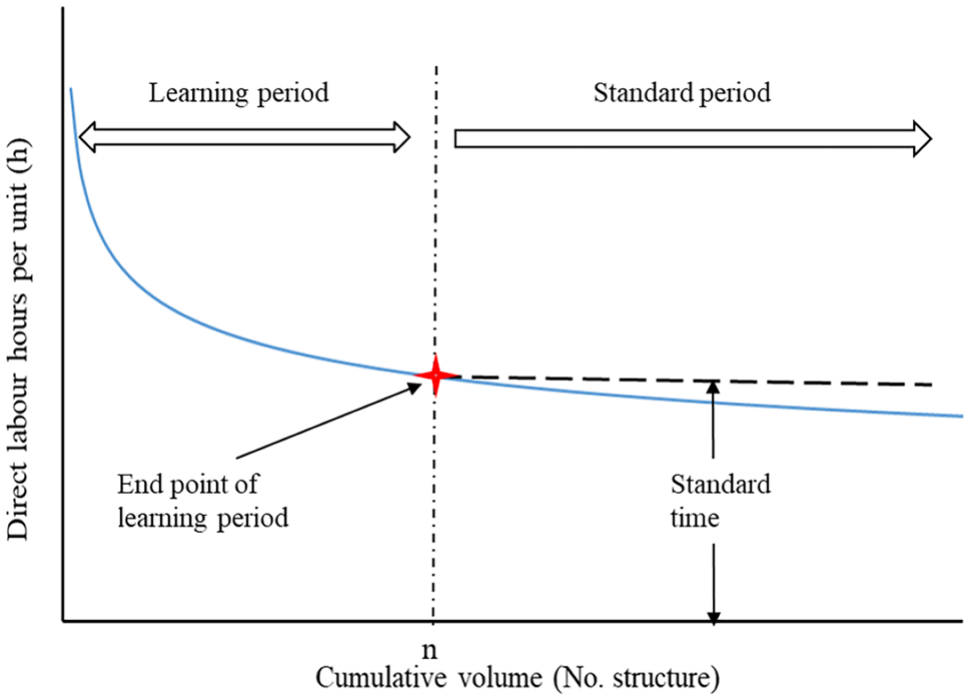
Standard learning curve (Krajewski et al. 2010)

Depending on the decline rate of labour hours, this production period can be divided into two periods — the learning period and the standard period, as shown in Fig. 3.

The learning period corresponds to the left segment of the learning curve, where the labour hours required to produce a unit product decrease rapidly as the number of products increases, implying that workers improve their skills quickly through multiple repetitions during this period. When the production quantity exceeds a specific value, *n*, it can be seen that although the labour hours required for unit production keep decreasing, the decrease rate becomes much lower. This means that the learner becomes proficient in this stage, and it is harder to increase production efficiencies further. It is worth noting that according to Eq. 21, the labour hours will decrease infinitely as the number of production increases, which is not practical. There must be a standard time, the minimum time required to produce a unit by a fully skilled worker. Therefore, the first unit produced at the standard time is defined as the endpoint of the learning period, and this standard time, ${y}_{s}$, equals the value estimated using the learning curve at the endpoint, *n* (Eq. 23):

23 $${y}_{s}=a{n}^{b}$$

It means the labour hours used to manufacture door is a constant (${y}_{s}$) when producing the *n*th product.

In the present study, a method is developed accordingly to calculate the value *n*. The decrease rate, $r$, which is the difference of direct labour hours between the *m*th and (*m* + 1)$th$ unit product, can be calculated (Eq. 24):

24 $$r=\frac{{y}_{i}-{y}_{i+1}}{{y}_{i}}$$

where ${y}_{i}$ and ${y}_{i+1}$ are the direct labour hours for the i*th* and (i + 1)*th* unit product, respectively. The value *r* decreases as the production quantity increases. It is assumed that the standard time has been reached when the *r* value reduces below a certain value ($\varepsilon$) (Eq. 25):

25 $$r\le \varepsilon$$

by solving Eqs. (18), (21) and (22), the *x* value range is calculated as shown in Eq. 26:

26 $$x\ge \frac{1}{{\left(1-\varepsilon \right)}^{1/b}-1}$$

Therefore, $n$ can be calculated to be the smallest integer that satisfies the inequality, as shown in Eq. 27:

27 $$n=\left\lceil\frac{1}{{\left(1\,-\,\varepsilon \right)}^{1/b}-1}\right\rceil=min \;\left\{\left.x\;\in \;Z\right| x\ge \frac{1}{{\left(1\,-\,\varepsilon \right)}^{1/b}-1}\right\}$$

where $$\varepsilon \ge \frac{{\mathrm{Y}}_{i}\,-\,{\mathrm{Y}}_{i\,+\,1}}{{\mathrm{Y}}_{i}}$$.

#### Cost inventory data and sources

The main costs and data sources are listed in Appendix Table iv. The material and energy consumptions are based on the industry data listed in Appendix Tables v, vi for the composite and aluminium doors, respectively. Both material and consumable costs are included in the total material cost. The price of the material varies with its availability and market demand in different years. As there is a lack of preliminary data, the Federal Reserve Economic Data source (Federal Reserve Bank of St. Louis 2021) for rolling forward historical costs to present value in 2020 is considered in Eq. 28:

28 $${Euros}_{base \;year}={Euros}_{data\; year}\times \frac{{Price \;Index}_{base\; year}}{{Price\; Index}_{data \;year}}$$

where the base year is 2020 and the data year is the year of publication for a dataset citation.

The main assumptions are (I) the manufacturing process will not vary in the high-volume manufacturing of door production and (II) the learning curve slope used in the analysis is 87% (Mislick and Nussbaum 2015; Matrone and Ascione 2018).

## Results and discussion

### Life cycle assessment

In this research, the global warming potential (GWP) in the calculation of both case studies is used, as the GWP is the most used impact indicator in LCA (van der Harst et al. 2016).

**Fig. 4:**
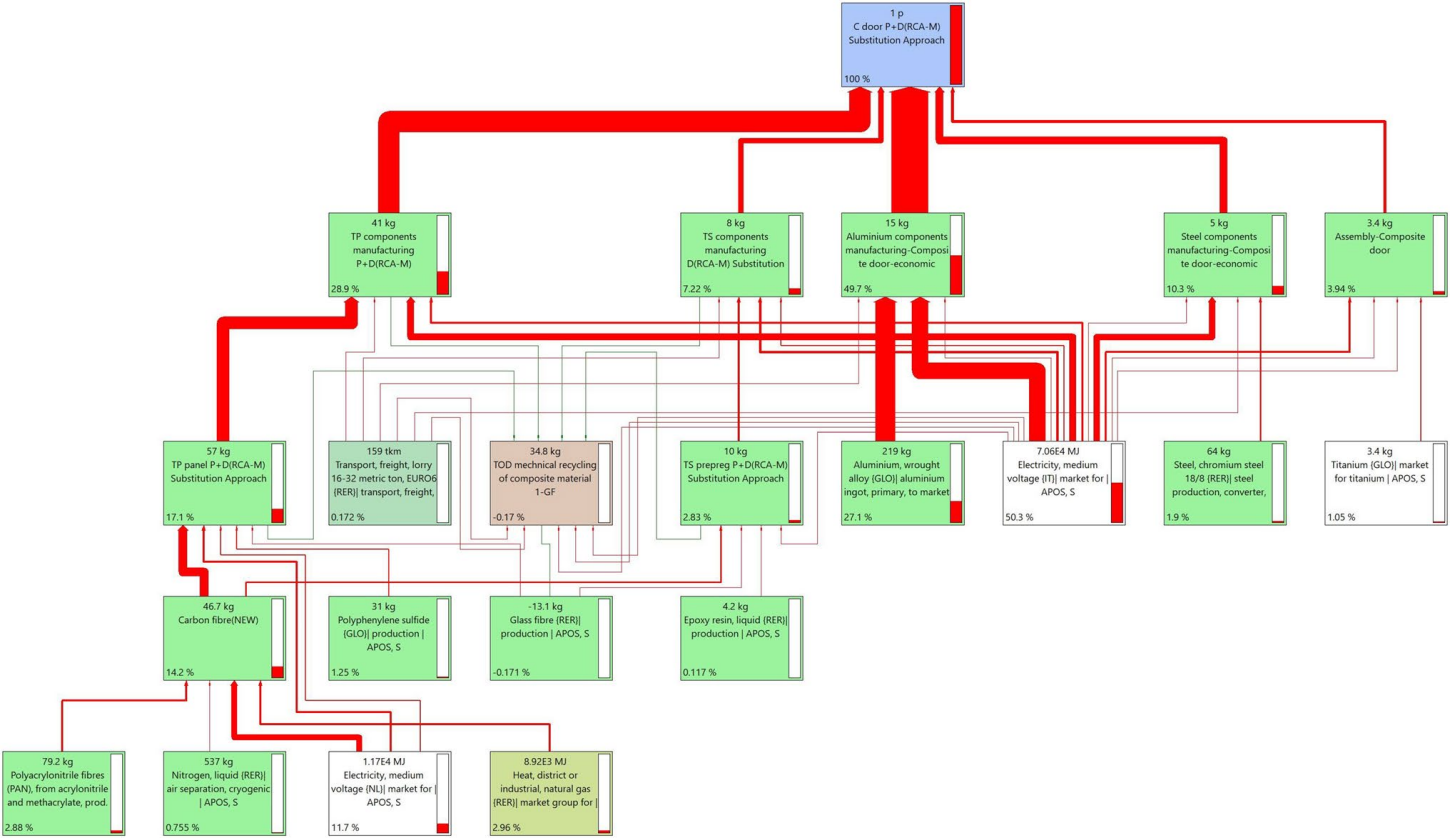
The network structure for the global warming potential of the production of the composite door based on impact 2002+ method

It can be seen from Fig. 4 that among the sources contributing to GHG emissions, energy consumption was identified as the hotspot, contributing approximately 65% of overall GHG emissions. Aluminium alloy production accounted for the second largest portion, 27.1% of the overall emission, followed by the production of PAN, SS, Ti, PPS and nitrogen, accounting for 2.88%, 1.90%, 1.05%, 1.25% and 0.76%, respectively. In contrast, the production of the remaining sources, such as epoxy resin and transportation, had fewer impacts (less than 0.2%), which could be neglectable. It should be noted that the environmental impacts of waste treatment and the production of glass fibre are negative. This is mainly due to the ‘avoided burden’ credits from the recycled glass fibre in the waste management processes leading to the assumed avoidance of the need for the production of virgin glass fibre materials. There is no doubt that recycling with recovery value from composites can help the environmental performance improvements slightly.

**Fig. 5:**
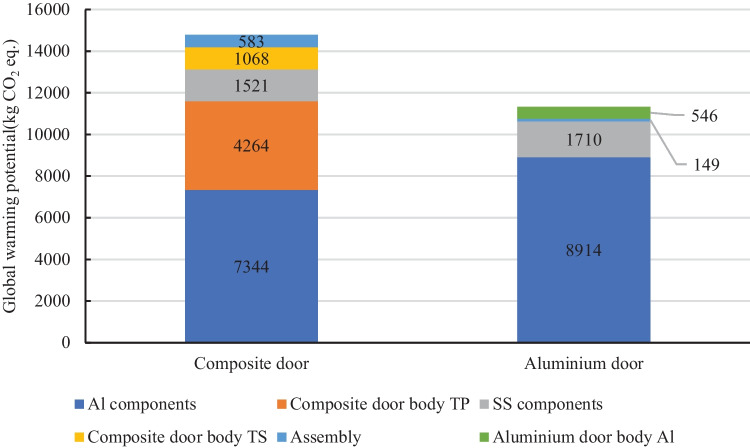
The comparison of the global warming potential caused by producing one FU of aircraft door associated with all the main production processes of the door based on impact 2002+ method

Figure 5 shows GWP score environmental impact for the two manufacturing scenarios considered in this study. The GHG emission contribution of each process to produce one FU of aircraft doors was presented. For the composite door, the Al components’ manufacturing process caused the highest GHG emissions (7344 kg CO_2_ eq.), accounting for 50% of the total GHG emissions, and this is around two times higher than that caused by TP components’ manufacturing process (around 29%). The SS and TS components’ manufacturing processes contributed lower GHG emissions to the total environmental performance at 1521 kg CO_2_ eq. (around 10%) and 1068 kg CO_2_ eq. (around 7%), respectively. The percentage contributed by the assembly process was the lowest (583 kg CO_2_ eq.), at about 4%. A similar trend is found in aluminium door production. The Al components for the door fittings and mechanism had the highest GHG emissions (approximately 8914 kg CO_2_ eq.). The aluminium door body manufacturing and SS components’ manufacturing processes contributed lower GHG emissions, and the assembly process contributed the lowest GHG emissions. Comparing the impacts caused by producing Al components for two aircraft doors, the aluminium door produced 1570 kg CO_2_ eq. more GHG emissions. These differences in the door fittings and door mechanism are likely due to the difference in design for compromising the configuration of two doors. The GWP caused by the assembly of the aluminium door were 434 kg CO_2_ eq., less than that of composite counterparts due to the lower energy intensity of mechanical fasteners used in the aluminium door assembly compared to the higher energy consumption of induction welding of the composite door assembly. The total impacts of SS components were similar in comparison to the composite door (1710 versus 1,521 kg CO_2_ eq.). In contrast, the main differences between the two doors to the GWP were directly related to the door body production. That is the main part where the standard metallic materials are replaced by composites. Interestingly, it can be seen that the composite door body had GHG emissions around 877% greater than the aluminium door body.

It is worth noting that the weight of the composite door body is higher than the aluminium door body, which conflicts with the general expectation that the usage of composite is lower the weight. These differences in the door body are due to the differences in design between the two doors. The composite door modular design means the body structure has more built-in components, and the design engineers adopt more conservative safety factors than those used for the metallic door at the present state of the art in composite door production. On the other hand, aluminium door production uses chemical milling and low thickness allowed by sheet metal technology, resulting in much lower weight adapted in the door production. In this case, an additional comparison has been carried out by assuming the weight of the optimised composite door body is 80% of the conventional one, which is a typical weight saving of 20% reported by some literature (Liu 2013; Timmis et al. 2014) to produce a structure replacement with that of aluminium alloy. This assumption aims to compare the results of this work against the results from the previous LCA studies for the substitution of aluminium by composite materials in the aircraft sector to validate the model and its associated data. The comparison results can be found in Table 2.

**Table 2:** The global warming potential of door body production during the production stage

Impact category	Aluminium door body	Composite door body	Composite door body weight saving 20%
Global warming potential (kg CO_2_ eq.)	546	5332	1173

Comprising the two composite doors body (the proposed door vs the anticipated weight-saving ones), the GHG emissions for the anticipated composite door were significantly reduced; the decrease was up to 78%. The greater the weight reduction, the lower the material required during manufacture. However, even if the weight of the novel door body was reduced after optimising the structure to reach the 20% weight savings as the aluminium door body, the composite door body could not achieve a similar environmental performance as the metal door body did. Similar LCA results had been reported in the literature (Scelsi et al. 2011; Timmis et al. 2015; Vieira and Bravo 2016), which showed that carbon fibre composites performed worse than the industry standards of aluminium in the production process. This could be explained by the fact that a large amount of energy is consumed in carbon fibre production, i.e. the high energy intensity of vCFs’ production (Hann Chua et al. 2015; Sunter et al. 2015). Under such assumptions, if the use stage is considered as an extension of the current modelling work, the composite door body will have improved impacts than its metallic counterparts when the weight-induced fuel consumption model is used in the flight operation as discussed in the literature (Scelsi et al. 2011). Then there will be a minimal travel distance calculated to reach an environmental impact break-even to show the benefits from the implementation of lightweight structures.

### Life cycle costing

#### Cost estimation for the unit door

**Fig. 6:**
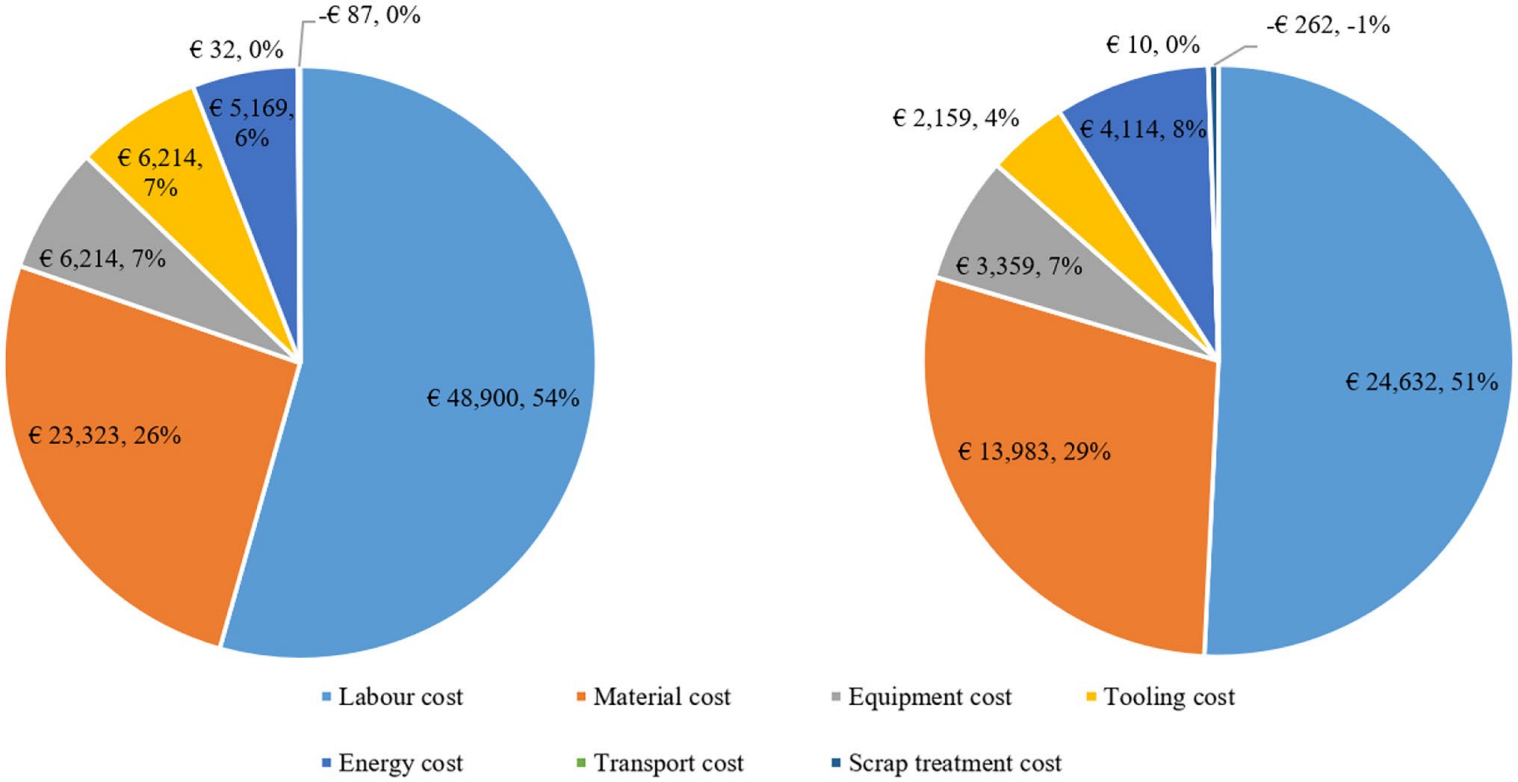
The contribution of each cost category in the production of the FU of aircraft door: the composite door (left panel) and the aluminium door (right panel)

The total costs (sum of all sub-costs for each production process) to produce a single composite door and aluminium door were estimated to be €89,765 and €47,996, respectively. The contribution of each cost category is given in Fig. 6.

It shows that the most significant cost contributions are labour cost at 54%, followed by material cost at 26% of the total cost. The equipment, tooling and energy only account for 7%, 7% and 6% of the total cost, respectively, while the proportion of the scrap treatment cost to the total cost is 0.2%. In terms of aluminium door production, similar to composite door production, the labour cost had the highest cost at around 51% of the total cost, followed by material cost (at €13,983, 29%). The energy, equipment and tooling costs contributed a moderate cost (between 4 and 8% of total cost), while the transport and the scrap treatment costs were negligible. The findings show a 46% lower LCC for aluminium door production compared to those composite door production. The LCC of each cost parameter was higher for the composite door production compared to the aluminium door.

The cost model was validated during a project meeting with industry specialists. The values and the formulas considered and obtained were discussed during the meeting. They had agreed on the achievement of model development, which meets their needs. The production cost result was then compared to findings in the literature. As no composite doors are available in the market, some other aircraft products’ manufacturing costs are compared for reference in Table 3.

**Table 3:** Relative cost comparison for aircraft products

**Product name**	**Material cost**	**Energy cost**	**Labour cost**	**Consumables**	**Shipping cost**	**Tooling cost**	**Recycling cost**	**Equipment cost**	**Facility**	**Source**
Frame	29%	-	51%	7%	13%	-	-	-	-	Matrone and Ascione (2018)
Helicopter’s canopy	19%	0	81%	-	-	-	0	-	-	Katsiropoulos et al. (2019)
Stringer	38%	-	38%	-	-	14%	10%	-	-	Hagnell and Åkermo (2015)
Skin panel	59%	1%	19%	-	-	2%	-	9%	10%	Shehab et al. (2013)

Although the cost elements considered in each case in Table 3 are different, the majority of the total manufacturing costs for the composite door being for labour and material is consistent with the dominance of the costs for the components. Notably, for skin panel production (Shehab et al. 2013), the labour cost at only 19% of the total manufacturing cost is relatively low due to China’s lower labour rate and relatively higher raw material price (Ma 2011).

#### Cost estimation for mass production

The price to produce a single door will be reduced as the production volume increases. This is because the learning curve indicates that the direct labour hours will decrease. In order to estimate the standard time of producing future units, the slope of 87% is used to establish the learning curve. The endpoint is determined following the criteria that the learning period ends when $r$ reduces below 5‰. Based on this, the value of $n$ is calculated to be 40 according to Eq. 24. Therefore, the labour hours required to produce each door will be constant from the 40th onwards and equal to that needed to produce the 40^th^ door. It was calculated that the labour hours were halved when entering the standard period (when the volume exceeds 40), and then the total cost for a single door was decreased by about 29% accordingly, to around €64,248.

**Fig. 7:**
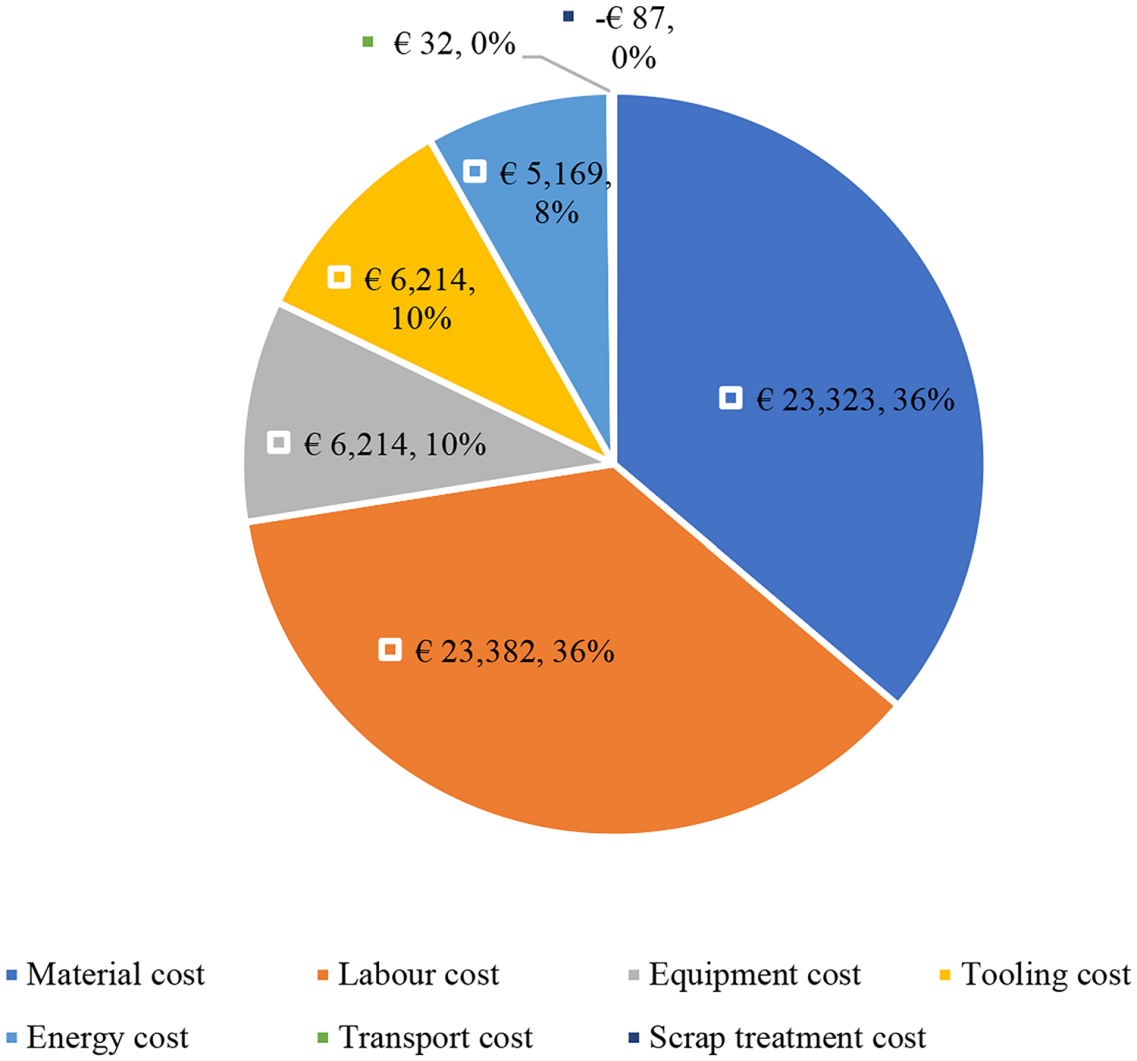
The contribution of each cost category in the production of the 40th composite door

The contribution of each category is analysed again, as shown in Fig. 7. It can be seen the contribution of labour cost was significantly reduced from €48,900 to €23,382 (approximately 52% of reduction), and the value was nearly equal to that for the material.

Overall, the door production cost tends to decrease as the production volume increases. This specific analysis has identified the endpoint regarding the number of units produced before the learning period ends. The results show that after producing 40 doors, the door production cost is constant, and each unit price reduces by around 29% compared to the first unit. It should be noted that the reduction in unit cost is only attributed to the proficiency increase in worker skills. The effect of the economics of scale, which may further reduce the unit price, is not considered due to the lack of primary data.

### Eco-efficiency (EE) analysis

**Fig. 8:**
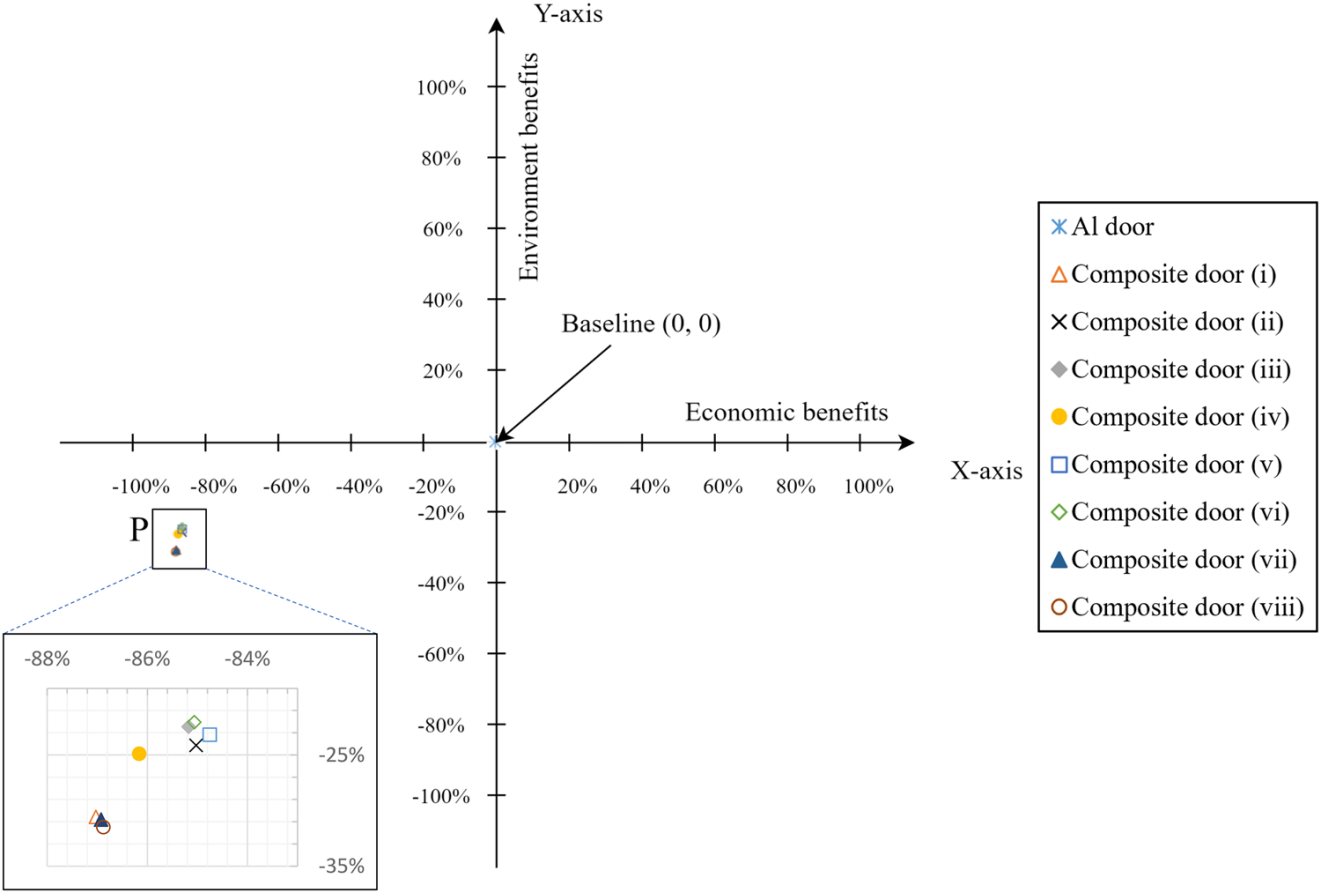
EE profiles of different scenarios for reference door (0, 0 on the axes) vs composite aircraft door with various waste composite recycling methods for (i) mechanical recycling, fibre replacement via substitution with correction factor or alternative material approach (R_CA_-M); (ii) pyrolysis recycling, fibre replacement via substitution with correction factor or alternative material approach (R_CA_-P); (iii) solvolysis recycling, fibre replacement via substitution with correction factor or alternative material approach (R_CA_-S); (iv) mechanical recycling, fibre replacement via substitution with equal quality approach (R_EQ_-M); (v) pyrolysis recycling, fibre replacement via substitution with equal quality approach (R_EQ_-P); (vi) solvolysis recycling, fibre replacement via substitution with equal quality approach (R_EQ_-S); (vii) landfill; and (viii) incineration

In order to perform an integrated analysis, the LCA and LCC results of the composite door are compared via the EE framework with that of the aluminium door. The GWP score result for environmental impacts is selected, and the performance of the composite door with the reference door systems is presented in the EE output in Fig. 8 at point *P*. It shows the composite door scenarios have a similar performance. In addition, the EE profile shows that the composite-based doors are not preferable to the aluminium-based alternative in either their environmental or economic profile. Therefore, improvement actions are recommended if the composite door is to offer a competitive alternative to replace the conventional door. In spite of that, the former could still be deemed the best option among eight composite door scenarios based on the practitioner’s criteria regarding the weights of environment and economic metrics. In this research, it is assumed that the environment and economic metrics are equally important. Therefore, the weight factors *A* = 0.5 and *B* = 0.5 are used. The EE index is calculated accordingly, as shown in Table 4. The result indicates that scenario (vii), the solvolysis recycling with equal quality CF substitution, presents the best performance by far for all analysed alternatives considered.

**Table 4:** EE index ranking among the composite door with various waste composite recycling methods for (i) mechanical recycling, fibre replacement via substitution with correction factor or alternative material approach (R_CA_-M); (ii) pyrolysis recycling, fibre replacement via substitution with correction factor or alternative material approach (R_CA_-P); (iii) solvolysis recycling, fibre replacement via substitution with correction factor or alternative material approach (R_CA_-S); (iv) mechanical recycling fibre replacement via substitution with equal quality approach (R_EQ_-M); (v) pyrolysis recycling, fibre replacement via substitution with equal quality approach (R_EQ_-P); (vi) solvolysis recycling, fibre replacement via substitution with equal quality approach (R_EQ_-S); (vii) landfill; and (viii) incineration

Ranking	1	2	3	4	5	6	7	8
Alternative No.	Composite door (vi)	Composite door (iii)	Composite door (v)	Composite door (ii)	Composite door (iv)	Composite door (i)	Composite door (vii)	Composite door (viii)
EE index	− 0.536	− 0.538	− 0.540	− 0.546	− 0.555	− 0.588	− 0.589	− 0.592

**Fig. 9:**
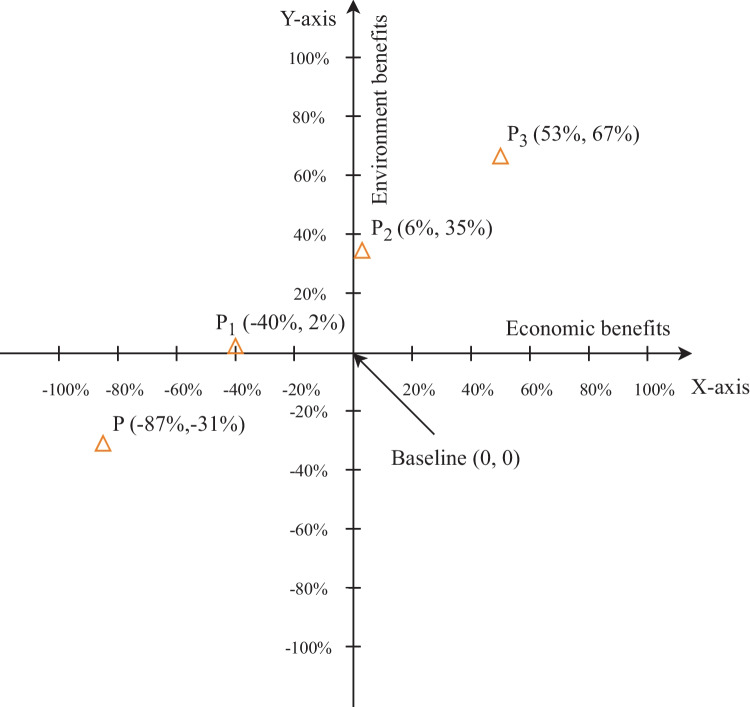
EE profiles of different scenarios for reference door (0, 0 on the axes) vs various weight reductions for the composite aircraft door: P: 0%; P_1_: 25%; P_2_: 50%; P_3_: 75%

In this case, the current composite structure system is based on a conservative design of multiple layers of laminates. Stakeholders would like to optimise laminates (e.g. reducing the thicknesses) to reduce the weight of the composite components in their future products, leading to lower material consumption. In order to evaluate the potential benefits of an optimised composite door and the reference door in terms of environmental and economic performances, a scenario analysis was conducted on the possibility of design and production changes in the weight reduction of the composite door. For the composite door with composite waste recycling via mechanical method using substitution with correction factor approach (R_CA_-M) as an example, the data set used a reduction in weight of the composite door of 0%, 25%, 50% and 75%, termed scenario P (current composite door); scenario P_1_; scenario P_2_; and scenario P_3_, respectively. Figure 9 shows that scenario P achieved the worst EE solution for the composite door over the reference. Scenario P_1_ was better in terms of environmental performance but not cost, while P_3_ is an improvement on P_2_, and these two scenarios are all green EE solutions compared with the reference. In addition, the GHG emissions and costs associated with composite door production progressively decrease proportionally with weight reduction. Therefore, a weight reduction of 47% for the composite door was calculated to reach a cost below the aluminium door cost. Then the composite door would be slightly better in both environmental and economic performances than the reference door if the composite door was optimised and continuously improved in the future design while retaining all functionality.

### Sensitivity analysis

#### Potential influence of composite waste treatment routes

There are some uncertainties concerning the waste treatment of composites as these are still under development, and a universal method is not yet on a market scale (Li et al. 2016). The techniques include landfilling, incineration and mechanical, thermal and chemical recycling (Pakdel et al. 2021). In the present study, the cradle-to-gate scope of the LCA was extended to include the potential environmental impacts of composite scrap waste treatment routes for the composite door. Due to the uncertainty of markets for recovered carbon fibre (rCF) from mechanical recycling, pyrolysis recycling and solvolysis recycling methods, we consider several scenarios to check the results to the potential value as fibres’ substitute.

First, the fibres’ substitute was based on the correction factor or alternative material in the applications:

(i): Mechanical recycling and the fibre replacement via substitution with correction factor or alternative material approach (R_CA_-M): the rCFs were assumed to replace the same volume of virgin glass fibres in the reference (Meng et al. 2018a), and this is the default recycling method used in this research.
(ii): Pyrolysis recycling and the fibre replacement via substitution with correction factor or alternative material approach (R_CA_-P): to account for the physical changes of recycled fibres due to the recycling process and determine equivalent quantities of virgin fibres, variable fibre content and variable material thickness methods (Meng et al. 2018b) were derived from qualifying the ability of recycled material to achieve mechanical performance equivalent to virgin material. As the environmental impacts of CF replacement based on variable fibre content and material thickness methods are similar (Meng et al. 2018a), an average value of the CF replacement ratio of 0.91 was used in this recycling scenario, i.e. 1 kg pyrolysis rCFs could replace 0.91 kg vCFs to achieve the same quality.
(iii): Solvolysis recycling and fibre replacement via substitution with correction factor or alternative material approach (R_CA_-S). As the solvolysis process also generated less degraded fibres (Meng et al. 2018a), a fibre replacement ratio of 0.95 was used.

Second, three higher market value scenarios were presented based on the use of rCF to displace virgin carbon fibre (vCF) on a 1:1 ratio in composite applications:

(iv): Mechanical recycling and fibre replacement via substitution with equal quality approach (R_EQ_-M).
(v): Pyrolysis recycling and fibre replacement via substitution with equal quality approach (R_EQ_-P).
(vi): Solvolysis recycling and fibre replacement via substitution with equal quality approach (R_EQ_-S).

Last, the above six scenarios were also compared to the two conventional composite waste treatment methods:

(vii): Landfill.
(viii): Incineration.

It was assumed transport distance of 100 km to incineration or landfill and a distance of 250 km to recycling facilities according to the EeBGuide project (Gyetvai 2012). The input and output information of each tested composite waste treatment method and its respective original sources are shown in Appendix Tables vii–xii. In addition, the midpoint GWP impact scores were compared based on the eight scenarios for composite door production, as shown in Table 5.

**Table 5:** LCA results for the global warming potential of composite door production based on the disposal of waste composites via different waste treatment methods, including (i) mechanical recycling, fibre replacement via substitution with correction factor or alternative material approach (R_CA_-M); (ii) pyrolysis recycling, fibre replacement via substitution with correction factor or alternative material approach (R_CA_-P); (iii) solvolysis recycling, fibre replacement via substitution with correction factor or alternative material approach (R_CA_-S); (iv) mechanical recycling, fibre replacement via substitution with equal quality approach (R_EQ_-M); (v) pyrolysis recycling, fibre replacement via substitution with equal quality approach (R_EQ_-P); (vi) solvolysis recycling, fibre replacement via substitution with equal quality approach (R_EQ_-S); (vii) landfill; and (viii) incineration

Impact category	Composite door (i)	Composite door (ii)	Composite door (iii)	Composite door (iv)	Composite door (v)	Composite door (vi)	Composite door (vii)	Composite door (viii)
Global warming potential (kg CO_2_ eq.)	14,780	14,052	13,862	14,137	13,942	13,816	14,808	14,884

The incineration scenario had the highest impact on GWP due to the amount of CO_2_ released in burnt composite, followed by landfill; a reduction of around 100 kg CO_2_ eq. was achieved compared to composite combustion. The recycling processes generated fewer impacts than incineration and landfill as the recycling scenarios benefit greatly from material reuse, crediting the avoided raw material production and enabling the emissions from the recycling process to be absorbed. In terms of the comparison of two substitution approaches applied in each recycling method, the GWP results for the substitution with the alternative material method were higher compared to the substitution with equal quality when the same recycling methods were used in the analysis. It indicates that the great environmental GWP benefits obtained from the recovery process where benefits are replacing vCFs with rCFs at a 1:1 ratio. Mechanical recycling could achieve a GWP of 642 kg CO_2_ eq. per composite door by accounting for a 4.3% reduction by avoiding vCFs production instead of vGFs production. This is because of the higher energy intensity of vCFs than that of virgin glass fibre. Due to the difference between fibre replacement in pyrolysis recycling and solvolysis recycling with different substitution methods, the 100% CF replacement resulted in a GHG emissions reduction of 109 kg CO_2_ eq. and 47 kg CO_2_ eq. per composite door, respectively, in comparison with the CF replacement ratio of respective 0.91 and 0.95 in these two recycling methods. In terms of the comparison of the three composite recycling methods, GWP results also show that solvolysis recycling led to the best GHG emission performance in each recycling method, followed by the pyrolysis scenario and then the mechanical recycling scenario. Overall, the composite door production with the composites treatment by incineration caused the highest greenhouse gas (GHG) emissions, only 7% higher than the lowest GHG emissions in scenario (vi), where the composites waste is recycled by the solvolysis method. These eight waste treatment routes had very similar GHG emissions for composite door production; in other words, the GWP results in terms of different waste treatment routes had nearly negligible effects on the door production process.

Moreover, the recycling scenarios generated fewer impacts than incineration and landfill mainly because recycling benefited greatly from material reuse, crediting the avoided raw material production and enabling the emissions from the recycling process to be absorbed. Furthermore, 100% vCF replacement can achieve the largest GWP reductions. These GWP reductions demonstrate the improvement of recycling technology is required to minimise the degradation of material properties due to recycling.

Landfill is currently still a common end-of-life practice for composites. However, results reported in La Rosa’s research (La Rosa et al. 2021) and Meng’s research (Meng et al. 2018a) show that landfilling is not a desirable end-of-life option among all the scenarios. Our results were expected and can be considered a further confirmation of the consistency of the literature. In addition, advanced recycling processes, including pyrolysis (Meyer et al. 2009) and solvolysis (Liu et al. 2004) technologies, show high potential emission reductions as viable recycling routes because they can achieve high fibre recovery rates and largely maintain the mechanical properties of recovered CF. Our scenario analysis demonstrates the potential viability of processes that can achieve higher GWP reductions.

#### Influence of cost parameters

**Fig. 10:**
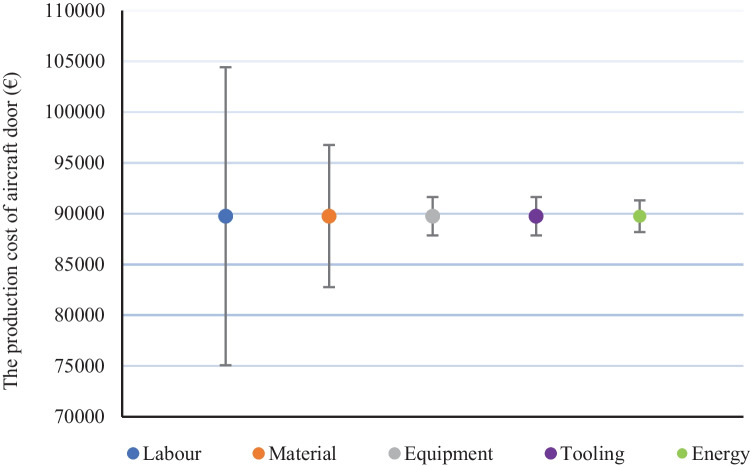
Local sensitivity analysis of cost parameters for composite aircraft door on the production phase

A cost sensitivity analysis was carried out to identify the cost parameters that significantly influence the production cost. Five out of seven parameters in the LCC model were investigated: material, labour, energy, equipment and tooling. The scrap treatment and transport costs were ignored due to their low contribution (i.e. only 0.1% and 0.04%, respectively). A local sensitivity analysis (Groen et al. 2017) was conducted, in which each parameter ranges from − 30% to 30% by changing one parameter at a time and keeping all others constant. The parameters show linear behaviour. So, the elasticity results for the most extreme sensitivities are presented. Their influence on production cost can be seen in Fig. 10.

Labour cost contributed the dominant impact on the door production cost (32.7%). The effect of the material cost was slightly lower than that of labour cost at 15.6%. Changes in the energy, equipment and tooling parameters resulted in negligible variations in door production cost (around 4.0%). This result suggests that reducing the labour cost is the most efficient way to reduce the total cost. The local sensitivity analysis is straightforward to interpret, but it only explores limited cost parameters to see how changes in input values affect the model outputs. It should be noted that this sensitivity analysis only separately considered the effect of cost parameters on the cost estimate, but not acted simultaneously on estimating the cost. Further analysis of the cost factors (i.e. unit price and labour rate) acting simultaneously on the cost estimate was presented in Sect. 4.5.

#### Influence of learning curve slope

**Fig. 11:**
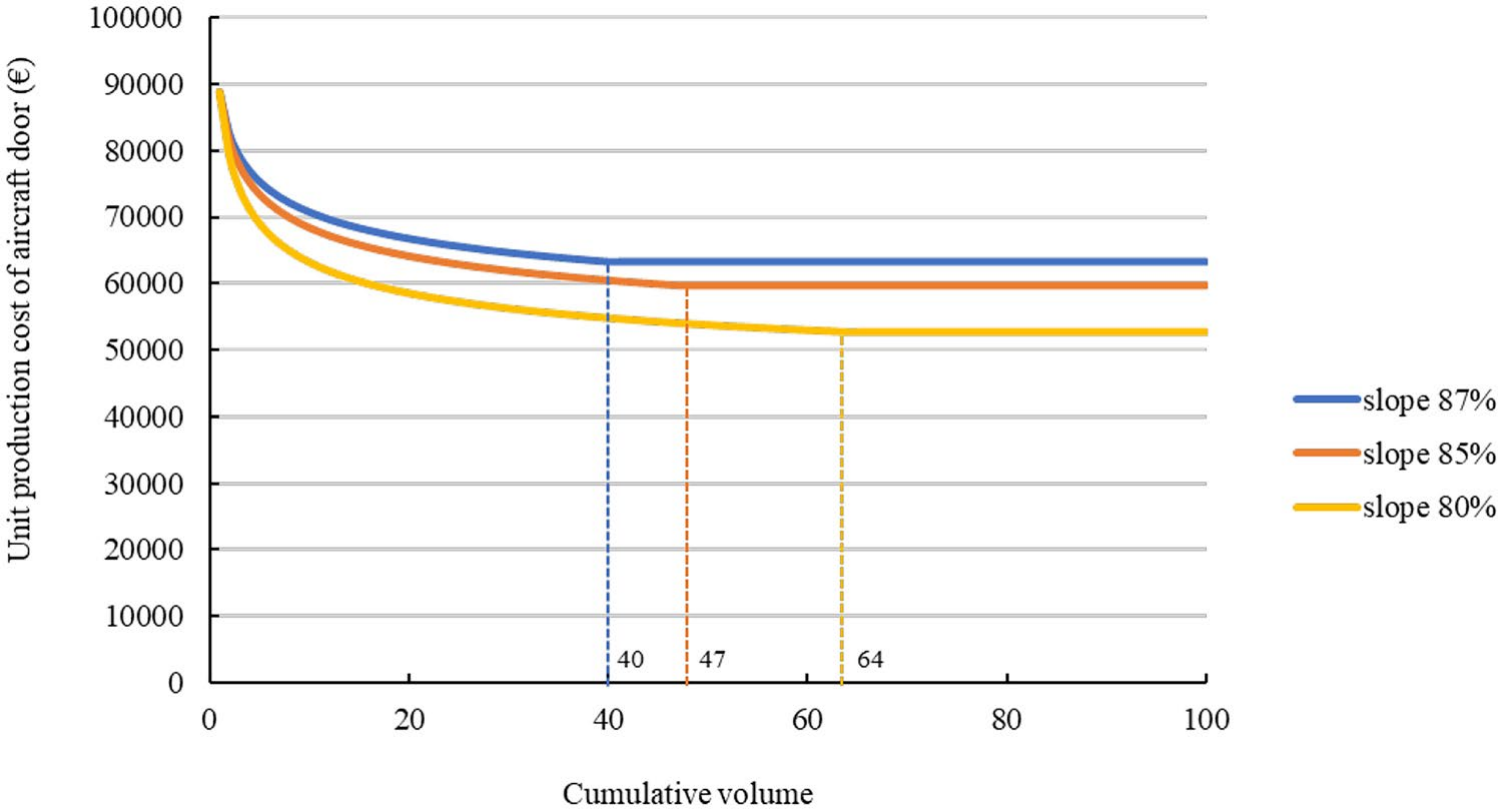
The unit production cost varies with increasing the volume based on different learning curve slopes

As the learning curve slope applied in the aircraft industry generally ranges from 80 to 87% (Hartley 1965; Moore 2015; Matrone and Ascione 2018), it is worth investigating the effect of slope value on the results. Therefore, three widely used values, 80%, 85% and 87%, are adopted in the present study, and the corresponding learning curves are plotted. The endpoint for each curve is also estimated based on the same criteria mentioned and plotted for comparison. In addition, the changes in unit production cost based on different learning curve slopes are also reported, as shown in Fig. 11. It shows that when the slope decreased, more units were required to be produced to achieve a standard period, i.e. the endpoints would increase (from unit 40 to 64). The unit production cost during the standard period was calculated related to the different slopes. The unit door production cost progressively reduced proportionally with the decrease of the learning curve slope. This result can be considered a validation of the applied production model. The unit cost dropped by nearly 16% (from €64,247 to €53,684), while the slope value decreased by 8% (from 87 to 80%). This result suggests that the learning curve slope is not a sensitive parameter in the cost analysis as it can only slightly affect the unit cost.

### Monte Carlo simulation

**Fig. 12:**
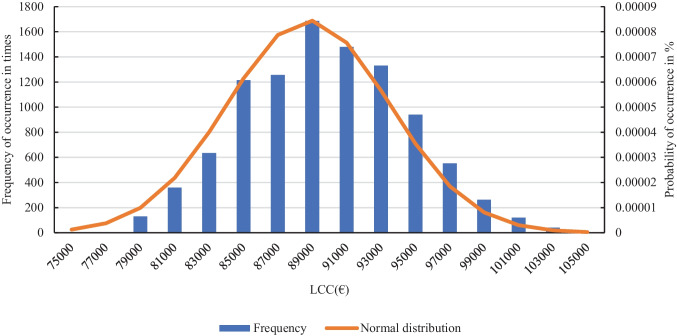
Probability distribution of door production cost with variables of labour rate and material unit costs

In this analysis, the top two contributing input cost parameters, labour (54% of production cost) and material (26% of production cost), are expressed through probabilistic distributions. The labour cost and material cost in the LCC model were determined by variations in labour rate and material unit price, respectively. The reason for further analysing these two factors was also driven by supply and market demand. Industry data correspond to information obtained before 2020. However, global supply chains and the price of raw materials have been widely affected since COVID-19 spread (Schmidt and Gelle 2021). Therefore, the effects of change in the material price are worth further analysis. In addition, labour rates, as independent input factors in each cost parameter, also warrant further consideration as they are subject to changes in the market forces. It is essential to explore further how these variations and uncertainties in the unit prices can influence the total LCC, considering the effect of factors acting simultaneously on the cost estimate. In this case, Monte Carlo simulation (MCS) was conducted. The direct labour rate of 63.5 €/h estimated by the industry suppliers was used in this research as the confidence in this was higher than in the literature values. Thus, the calculated labour factor of 93.98 €/h was used as the mode value, and the maximum value of 115 €/h and the minimum value of 88.9 €/h was chosen in the MCS (Bernet et al. 2002; Joshi 2018). Uncertainties associated with material costs may result from data accuracy or different geographical location. An average paid around 35% more for raw materials in 2021 compared to 2020 was reported by AlixPartners (Lampert and Singh 2021). Therefore, the average cost data from published data sources were used as the mode value. A triangle distribution of four main materials’ costs with a range of ± 35% variations was assumed to account for material uncertainties. According to the above assumptions, an MCS model was developed in an Excel spreadsheet. And then, the probability distribution under the MCS 10,000 runs for the composite door production cost can be calculated. Then the cost factors (i.e. unit price and labour rate) acting simultaneously on the cost estimate were presented in Fig. 12. It can be observed that the uncertainties associated with the variability in the five variables could lead to variations in the production cost of up to about 16% based on the minimum value of €78,361 and maximum value of €102,979. The study has shown the effectiveness of MCS in capturing the interactions between individual factors. The distributions can then be characterised statistically through mean and standard deviation and by providing the respective percentiles, as shown in Table 6. As can be seen, the mean value was €88,773 per door, which is higher than the production cost of the aluminium door; having a 2.5% chance of making a composite door was at €81,357 or less and €96,665 or more; having a 95% chance of making a door was between €81,357 and €96,665, i.e. the variation ranges from − 9.3 to 7.7%, when comparing the current composite concept (€89,765), featuring mechanical recycling of composites with fibre replacement via substitution with alternative material approach (R_CA_-M).

**Table 6:** Statistical description of results from Monte Carlo simulation

**Distribution**		**Percentiles**	
µ	σ	5%	95%
€ 88,773	€ 4721	€ 81,357	€ 96,665

The local sensitivity analysis is straightforward to interpret, but it only explores limited cost parameters to see how changes in input values affect the model outputs. The MCS further captures interactions between the individual factors. It should be kept in mind that the sensitivity and uncertainty analyses aim not to quantify every single type of uncertainty but to provide meaningful support for the decision-making process, highlighting potential key issues that may change the results and require further attention.

### Limitations and future developments

Currently, pyrolysis has reached the commercial scale (Pimenta and Pinho 2011; Greco et al. 2013), but solvolysis does not exist at a large scale yet (Khalil 2019). In this research, the pyrolysis and solvolysis processes were conducted on a laboratory scale to obtain the experimental data. It would have a lower energy consumption, resulting in a lower environmental footprint, if the same recycling process could be developed for deployment at a commercial scale.

In addition, the quality loss in the recycled metal materials is not considered in this research, similar to the other metallic material studies (van der Harst et al. 2016; Markatos and Pantelakis 2022). Compared to virgin aluminium, recycled aluminium and steel might have lower quality due to the impurity of recycled material, including Si, Mg, Ni and Zn (Dubreuil et al. 2010; Gaustad et al. 2012; Koffler and Florin 2013). Therefore, it may be appropriate to consider quality loss for metal recycling as composite recycling did by using substitution methods. A future study might build on the identification of relevant data, such as market prices of recycled material or quality indicators for quantifying the quality loss of metallic material.

It is worth noting that we defined a single aircraft door as the functional unit, assuming both doors completed functional equivalence with the same life span. It was based on experience-based judgement and conservative estimation by experts. However, the composite door was supposed to have a longer service than its metallic counterpart because carbon fibre composite has no fatigue limit and high corrosion resistance (Krauklis et al. 2021). Our case is more complicated because the induction welding technique was applied to join the new door’s beams and skins. Therefore, some new issues, such as failure on the welded joints, could emerge during the aircraft operation stage. Future research could be focused on conducting more specific experiments on such technology and the state-of-the-art application in aircraft doors. The decision-making shall be made carefully to identify the most promising materials in looking at the overall trade-off impacts of the composite door versus the conventional door.

The ecoinvent database version 3.8 is in-built and offered by commercial LCA software SimaPro 9.3. However, the database is updated yearly, introducing new and updated datasets every time. So, life cycle impact assessment (LCIA) results could be expected to change for the case study as LCI databases evolve. For example, all electricity markets in the old version had been updated; this alone influenced the LCIA results of almost all processes. Therefore, it is crucial to provide clear descriptions of the background datasets, including the data source, database version, year of publication and regional information, in the assessment reports where users can review source documentation and additional information. It would make LCA studies fully transparent on the data to ensure that everybody can access the validity of the research and its conclusions. In addition, a dynamic LCA approach might have the potential to offer more accurate results by addressing the inconsistency of temporal data (Beloin-Saint-Pierre et al. 2020). Therefore, further studies could focus on developing a time-dependent LCA model to assess the inventory flows in real-time impact scores for any given time horizon.

## Conclusion

This study presents an integrated LCA and LCC framework for analysing the environmental and economic attributes of products. Novel mass and energy flow inventory data and system boundaries were developed and demonstrated for a cradle-to-gate case study of both composite and conventional aircraft doors’ production. This study shows that a process-oriented LCA coupled with an LCC is relevant and efficient in gaining a useful overall assessment, validated by project specialists.

LCA results highlight that energy consumption was the single most significant hotspot, and the composite door had increased environmental impacts on the composite door production compared to the baseline of the conventional aluminium door. The addition of various potential end-of-life treatments based on two substitution methods (substitution with correction factor or alternative material and substitution with equal quality) for the composite waste generated during the production were compared to two traditional waste treatment routes, incineration and landfill. The inclusion of different waste treatment routes had only negligible environmental impact effects on the whole door production process. In addition, findings suggest that the improvement of recycling technology is required to minimise the degradation of material properties due to recycling.

Concerning the costs, the most significant cost contribution for the unit door production was labour and producing the composite door incurred higher costs than its metallic counterparts. An estimation method for future door production is conducted using the learning curve theory, and a sensitivity analysis demonstrates that the learning curve slope has a minor influence on the LCC result. Sensitivity analysis and MCS were used to help to address data uncertainty in the LCC. Local sensitivity analysis obtained the ranking of key cost parameters. The MCS further explored the labour rate and material unit price factors acting simultaneously on the cost estimate. Results show that the uncertainties associated with the variabilities could lead to variations in the composite door production cost of up to about 16%. It should be kept in mind that the sensitivity and uncertainty analyses aim to provide meaningful support for the decision-making process rather than to quantify every single type of uncertainty, highlighting potential key issues that may change the results and require further attention.

In addition to supporting making decisions, a graphical visualisation was proposed to model the alternative (in this case, the composite door) against reference in terms of their integrated environmental and economic performances. The analysis demonstrates that it is possible for the composite door to compete successfully with the conventional aluminium door in terms of both environmental and economic performance if weight reduction is achieved for the composite components in future designs. The tool has proved to be useful in representing an EE comparison based on the integration of the LCA and LCC results of potential modifications to the composite door against the reference door. The strength of the proposed method is the simplicity and, at the same time, completeness of the analysis.

Researchers need to be more aware of the potential influence of the used database on the conclusions and assessments drawn from a study. A closer look at the background information of different LCI databases would unlock data for total transparency and support researchers alike in the assessment results. We recommend using the latest or raw data as much as possible when conducting life cycle analysis. Most importantly, the results should be interpreted based on actual conditions in addition to the assessment. It will provide decision-makers with more reliable, authentic and objective performance measurement insights, allowing them to make informed strategies and policies for further sustainability research and improvements.

## Supplementary Information

Below is the link to the electronic supplementary material.

Supplementary file1 (DOCX 506 KB)

## Data Availability

All data generated or analysed during this study are included in this published article and its supplementary information files.
