# Supplementary material for: A novel life cycle assessment and life cycle costing framework for carbon fibre-reinforced composite materials in the aviation industry
Source: Int J Life Cycle Assess. 2023 Mar 28;28(5):566–89. doi: 10.1007/s11367-023-02164-y (PMC10043863; doi:10.1007/s11367-023-02164-y)
Supplement: Supplementary file 1 — Supplementary file1 (DOCX 506 KB) [file 11367_2023_2164_MOESM1_ESM.docx]

# APPENDIX TABLE

TABLE i. The detailed description of composite door and aluminium door production

| **Composite door production description** | | | |
| --- | --- | --- | --- |
| **Manufacturing process** | **Part name in the structure** | **Raw material** | **Production procedure** |
| TP components manufacturing | Outer and inner skin, vertical and horizontal beams of the door | Toray Cetex TC1100 300J 5HS refers to carbon fibre PPS prepregs, where TC1100 is polyphenylene sulphide (PPS) thermoplastic resin reinforced by carbon fibre T300J: 54.5% of carbon fibre, 2% of glass fibre and 43.5% of PPS resin by weight | (1) All plies of prepregs are hand laid up on heated press plates  (2) The TP panel is cut into smaller blanks (3) The blanks are loaded onto a heating die, the mould couple applied and thermoformed under pressure in an Infrared oven for pressing (4) Parts are demoulded to be cooled at air temperature  (5) Parts are trimmed to form the shapes of the final components (6) Inspection are performed using the IntACom system |
| TS components manufacturing | Door pan | Solvay Cycom 977-2-34-24KIMS-196 Unidirectional Tape refers to carbon fibre epoxy prepregs, where IMS9772 is an epoxy resin reinforced by carbon fibre IMS in unidirectional tape: 62% of carbon fibre, 4% of glass fibre and 34% of epoxy resin by weight | (1) All prepregs are stacked atop each other to get the desired orientation and thickness on a mould (2) The four-step thermoset process is conducted. i) a bag is sealed on the top layer and out of the edge of the part; ii) a vacuum is applied to remove air and avoid wrinkles; iii) the excess resin is removed, and the mould is placed into the autoclave to cure; iv) demoulding is applied (3) The cured parts are trimmed to the final shape (4) Inspection are performed using the IntACom system |
| Al components manufacturing | Door handles, lock mechanisms and fittings | Aluminium Al7075 or Al2024 family | (1) The aluminium ingots are hot rolled  (2) Parts are machined using computer numerical control systems (CNC): i) the virtual representation of the parts in CAD software is designed; ii) translating the CAD files into code for machining; iii) the users create the milling machine path through dedicated software converted in computer numerical machine language operations; iv) the metallic parts are machined to the desired shape and size (3) Inspection are performed using ultraviolet backlight fluorescent |
| SS components manufacturing | Door handles, lock mechanisms and fittings | 18-8 stainless steel | The same as the Al components manufacturing above |
| Joining/assembly | Door |  | (1) TP components are joined together using induction welding (2) All the components are joined with rivets that fasten the assembly together after appropriate inspections |
| **Aluminium door production description** | | | |
| Aluminium door body manufacturing | Outer skin, beams and gussets | Aluminium Al7075 or Al2024 family | (1) Cutting: the raw material is cut to shape using a three-axis contouring machine (2) Machining: a four-axis machining centre and a high-speed five-axis machining centre conduct multiple-point cutting to further shape the workpiece  (3) Stamping: the final shapes are achieved by using a press brake machine. (4) Inspection: the final tests are performed to ensure integrity. |
| Al components manufacturing | Door handles, lock mechanisms and fittings | Aluminium Al7075 or Al2024 family | The same as the Al components manufacturing in composite door above |
| SS components manufacturing | Door handles, lock mechanisms and fittings | 18-8 stainless steel | The same as the Al components manufacturing in composite door above |
| Joining/assembly | Door |  | All the components are joined with rivets that fasten the assembly together after appropriate inspections |

TABLE ii. Material and process datasets selected for composite door.

| **Material/process** | **Database** | **Source** |
| --- | --- | --- |
| PAN fibres | Polyacrylonitrile fibres (PAN), from acrylonitrile and methacrylate, prod. mix, PAN w/o additives EU-27 S | ELCD v3.2, 2018 |
| Nitrogen | Nitrogen, liquid {RER}\| air separation, cryogenic \| APOS, S | Ecoinvent 3.8, 2021 |
| Glass fibre | Glass fibre {RER}\| production \| APOS, S | Ecoinvent 3.8, 2021 |
| PPS resin | Polyphenylene sulfide {GLO}\| production \| APOS, S | Ecoinvent 3.8, 2021 |
| Epoxy resin | Epoxy resin, liquid {RER}\| production \| APOS, S | Ecoinvent 3.8, 2021 |
| Al alloy | Aluminium, wrought alloy {GLO}\| aluminium ingot, primary, to market \| APOS, S | Ecoinvent 3.8, 2021 |
| Steel alloy | Steel, chromium steel 18/8 {RER}\| steel production, converter, chromium steel 18/8 \| APOS, S | Ecoinvent 3.8, 2021 |
| Titanium rivets | Titanium, primary {GLO}\| production \| APOS, S | Ecoinvent 3.8, 2021 |
| Road transport | Transport, freight, lorry 16-32 metric ton, EURO6 {RER}\| transport, freight, lorry 16-32 metric ton, EURO6 \| APOS, S | Ecoinvent 3.8, 2021 |
| Dutch electricity | Electricity, medium voltage {NL}\| market for \| APOS, S | Ecoinvent 3.8, 2021 |
| Italian electricity | Electricity, medium voltage {IT}\| market for \| APOS, S | Ecoinvent 3.8, 2021 |
| Heat | Heat, district or industrial, natural gas {RER}\| market group for \| APOS, S | Ecoinvent 3.8, 2021 |

TABLE iii. Material and process datasets selected for aluminium door.

| **Material/process** | **Database** | **Source** |
| --- | --- | --- |
| Al alloy | Aluminium, wrought alloy {GLO}\| aluminium ingot, primary, to market \| APOS, S | Ecoinvent 3.8, 2021 |
| Steel alloy | Steel, chromium steel 18/8 {RER}\| steel production, converter, chromium steel 18/8 \| APOS, S | Ecoinvent 3.8, 2021 |
| Road transport | Transport, freight, lorry 16-32 metric ton, EURO6 {RER}\| transport, freight, lorry 16-32 metric ton, EURO6 \| APOS, S | Ecoinvent 3.8, 2021 |
| Italian electricity | Electricity, medium voltage {IT}\| market for \| APOS, S | Ecoinvent 3.8, 2021 |

TABLE iv. Cost data summary of the material, scraps treatment and revenue. ^1^

| **Item** | **Cost (€)** | **Unit basis** | **Price in 2020** | **Data sources** |
| --- | --- | --- | --- | --- |
| CF/PPS prepreg | 140 | /kg | 153.6 | (Long 2005) |
| CF/epoxy prepreg | 91 | /kg | 109.4 | (Long 2005) |
| Al alloy | 30.8 | /kg | 30.3 | (Zhai 2012) |
| Stainless steel | 18.5 | /kg | 13.6 | (Zhai 2012) |
| Rivets | 73.8 | /kg | 54.3 | (Zhai 2012) |
| Energy rate | 0.2 | /kWh | 0.2 | (Global Petrol Prices 2020) |
| Labour rate | 60 | /h | 63.5 | (Matrone and Ascione 2018) |
| Scrap Al | -0.5 | /kg | 0.6 | (Pinto 2017) |
| Scrap SS | -0.5 | /kg | 0.4 | (Pinto 2017) |
| glass fibre | 0.76 | /kg | 0.82 | (X. Li, Bai, and McKechnie 2016) |
| Filler (calcium carbonate) | 0.22 | /kg | 0.24 | (X. Li, Bai, and McKechnie 2016) |
| Mechanical recycling process no avoided materials | 3.56 | /kg | 3.56 | (Hedlund-åström 2005; X. Li, Bai, and McKechnie 2016) |
| Road transport | 0.15 | /t-km | 0.2 | (Schade et al. 2006) |
| Air transport | 0.75 | /t-km | 1.11 | (Schade et al. 2006) |

^1^ All cost data collected from literature has been converted to 2020 EU euro; Positive transactions represent expenses (costs) for door manufacturers; negative transactions represent revenues (benefits).

TABLE v. Industry data summary of energy, labour, raw material and scraps associated with the production of the composite door.

| **Manufacturing process** | | ***W* (kg)** | ***W_S_* (kg)** | ***ρ* (kWh/kg)** | ***P* (kW)** | ***T_M_* (h)** | ***R*_Lay_ (kg/h)** | ***T_L_* (h)** |
| --- | --- | --- | --- | --- | --- | --- | --- | --- |
| TP components' manufacturing | CF/PPS prepreg production | 71.3 | 0 | 11.1 | - | - | 1.2 | - |
|  | TP panel production | 57 | 14.3 | 6.1 | - | - | - | - |
|  | Preparing | 57 | 0 | - | - | - | - | 20 |
|  | Cutting | 41 | 16 | - | 5 | 9 | - | 14 |
|  | Thermoforming |  |  | - | 87 | 40 | - | 60 |
|  | Trimming/Drilling |  |  | - | 3 | 15 | - | 23 |
|  | Inspection | 41 | 0 | - | 22 | 45 | - | 45 |
| TS components' manufacturing | CF/epoxy prepreg  production | 12.5 | 0 | 11.1 | - | - | - | - |
|  | Store in refrigerator | 12.5 | 0 | - | 2 | 336 | - | - |
|  | Hand lay-up | 10 | 2.5 | - | - | - | 1.2 | - |
|  | Thermoset process | 8 | 2 | - | 80 | 8 | - | 20 |
|  | Inspection | 8 | 0 | - | 22 | 18 | - | 18 |
| Al components' manufacturing | Machining | 300 | 285 | - | 120 | 100 | - | 25 |
|  | Inspection | 15 | 0 | - | 3 | 20 | - | 30 |
| SS components' manufacturing | Machining | 100 | 95 | - | 120 | 42.5 | - | 10 |
|  | Inspection | 5 | 0 | - | 3 | 3.5 | - | 5.5 |
| Assembly | Induction welding | 69 | 0 | - | 12.5 | 84 | - | 134 |
|  | Mechanical fastenings | 72.4 | 0 | - | 3 | 26 | - | 46 |

TABLE vi. Industry data summary of energy, labour and raw material associated with the production of the aluminium door.

| **Manufacturing process** | | ***W* (kg)** | ***W_S_* (kg)** | ***P* (kW)** | ***T_M_* (h)** | ***T_L_* (h)** |
| --- | --- | --- | --- | --- | --- | --- |
| Aluminum door body's manufacturing | Preparing | 27.5 | 0 | - | - | 11 |
|  | Cutting | 13.5 | 14 | 5 | 5 | 7.5 |
|  | Machining |  |  | 12 | 9 | 16 |
|  | Stamping |  |  | 4 | 1 | 1 |
|  | Inspection | 13.5 | 0 | 3 | 2 | 3 |
| Al components' manufacturing | Machining | 367.5 | 349.1 | 120 | 120 | 30 |
|  | Inspection | 18.4 | 0 | 3 | 25 | 37.5 |
| SS components' manufacturing | Machining | 115 | 109.2 | 120 | 47.5 | 11.3 |
|  | Inspection | 5.8 | 0 | 3 | 4.5 | 6.8 |
| Assembly | Door body jointing | 13.5 | 0 | 3 | 70 | 92 |
|  | Inspection | 13.5 | 0 | - | - | 16 |
|  | Final assembly | 40.9 | 0 | 3 | 30 | 30 |

TABLE vii. Inventory data for mechanical recycling of 1kg composite waste, selected from Ecoinvent 3.8.

| Process | Item | Amount | Unit | Sources |
| --- | --- | --- | --- | --- |
| Input | Composite | 1 | kg | (Meng, Pickering, and Mckechnie 2018) |
|  | Electricity, medium voltage {IT}\| electricity voltage transformation from high to medium voltage \| APOS, S | 0.27 | MJ |  |
| Output | rCF | 0.24 | kg |  |
|  | Fine powder | 0.19 | kg |  |
|  | Coarse fraction | 0.57 | kg |  |

TABLE viii. Inventory data for pyrolysis recycling of 1kg composite waste, selected from Ecoinvent 3.8.

| Process | Item | Amount | Unit | Sources |
| --- | --- | --- | --- | --- |
| Input | Composite | 1 | kg | (Pakdel et al. 2021; Meng, Pickering, and Mckechnie 2018; Krauklis et al. 2021) |
|  | Electricity, medium voltage {IT}\| electricity voltage transformation from high to medium voltage \| APOS, S | 13.7 | MJ |  |
|  | Natural gas, burned in gas motor, for storage {GLO}\| market for \| APOS, S | 23.8 | MJ |  |
| Output | rCF | 0.62 | kg |  |
|  | Char | 0.14 | kg |  |
|  | Waste water | 0.03 | kg |  |
|  | Pentane | 0.03 | kg |  |
|  | Benzene | 0.03 | kg |  |
|  | Ethyl acetate | 0.1 | kg |  |
|  | Methanol | 0.04 | kg |  |
|  | Carbon dioxide | 0.003 | kg |  |
|  | Carbon monoxide | 0.001 | kg |  |
|  | Methane | 0.003 | kg |  |
|  | Ethane | 0.002 | kg |  |
|  | Propane | 0.001 | kg |  |
|  | Cyclopropane | 0.002 | kg |  |

TABLE x. Inventory data for solvolysis recycling of 1kg composite waste, selected from Ecoinvent 3.8.

| Process | Item | Amount | Unit | Sources |
| --- | --- | --- | --- | --- |
| Input | Composite | 1 | kg | (Angela Daniela La Rosa et al. 2021; Angela D. La Rosa et al. 2018) |
|  | Electricity, medium voltage {IT}\| electricity voltage transformation from high to medium voltage \| APOS, S | 10.81 | MJ |  |
|  | Acetic acid, without water, in 98% solution state {RER}\| acetic acid production, product in 98% solution state \| APOS, S | 0.51 | kg |  |
|  | Water, decarbonised, at user {RER}\| water production and supply, decarbonised \| APOS, S | 10.70 | kg |  |
|  | Sodium hydroxide, without water, in 50% solution state {GLO}\| market for \| APOS, S | 0.34 | kg |  |
| output | rCF | 0.63 | kg |  |
|  | Polycarbonate {RER}\| production \| APOS, S | 0.35 | kg |  |
|  | Waste water | 10.70 | kg |  |
|  | Waste plastic, mixture {Europe without Switzerland}\| treatment of waste plastic, mixture, sanitary landfill \| APOS, S | 0.01 | kg |  |

TABLE xi. Inventory data for landfill of 1kg composite waste, selected from Ecoinvent.

| Process | Item | Amount | Unit | Sources |
| --- | --- | --- | --- | --- |
| Input | Composite | 1 | kg | (Witik et al. 2013; Howarth, Mareddy, and Mativenga 2014) |
|  | Electricity, medium voltage {IT}\| electricity voltage transformation from high to medium voltage \| APOS, S | 0.27 | MJ |  |
| Output | Waste plastic, mixture {Europe without Switzerland}\| treatment of waste plastic, mixture, sanitary landfill \| APOS, S | 1 | kg |  |

TABLE xii. Inventory data for incineration of 1kg composite waste, selected from Ecoinvent 3.8

| Process | Item | Amount | Unit | Sources |
| --- | --- | --- | --- | --- |
| Input | Composite | 1 | kg | (Liu 2013) |
|  | Electricity, medium voltage {IT}\| electricity voltage transformation from high to medium voltage \| APOS, S | 32 | MJ |  |
| output | Energy | 10.51 | MJ |  |
|  | Waste water | 0.42 | kg |  |
|  | Xylene | 0.04 | kg |  |
|  | Styrene | 0.03 | kg |  |
|  | Dimethyl formamide | 0.01 | kg |  |
|  | Sodium hydroxide | 0.05 | kg |  |
|  | hydrogen cyanide | 0.05 | kg |  |
|  | 1-Butene C4H8 | 0.05 | kg |  |
|  | 1,2-Butanediol | 0.05 | kg |  |
|  | Bromine | 0.05 | kg |  |
|  | Acetone | 0.05 | kg |  |
|  | Acetonitrile | 0.05 | kg |  |
|  | Cyclopentadiene | 0.05 | kg |  |

# APPENDIX FIGURE

Using different colour blocks distinguishes the main production processes.


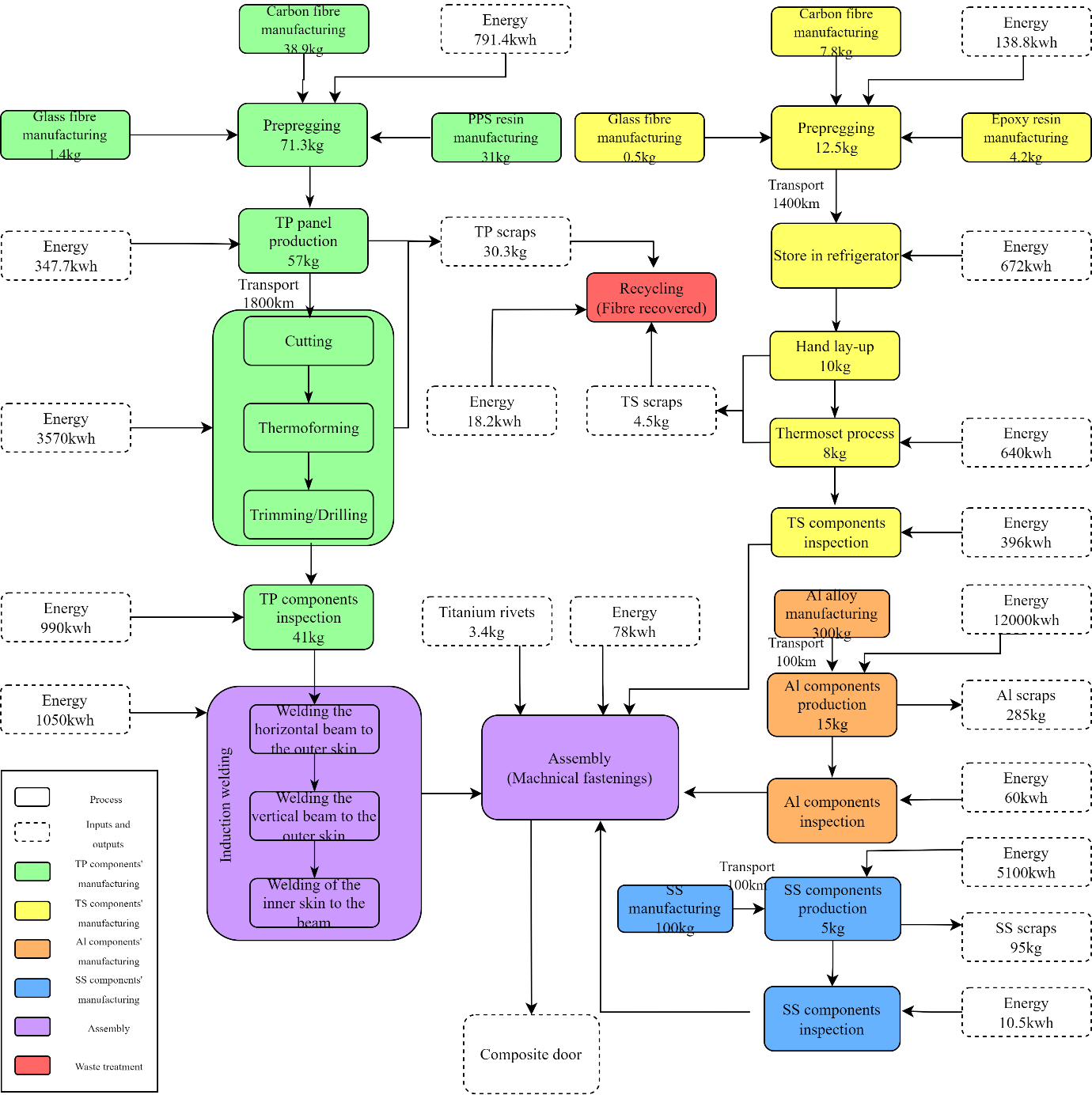


FIGURE i. The material and energy flows of the composite door’s manufacturing. The block colour indicates the type of each production activity: Green – TP components’ manufacturing, yellow – TS components’ manufacturing, orange – Al components’ manufacturing, blue – SS components’ manufacturing, purple – Assembly.


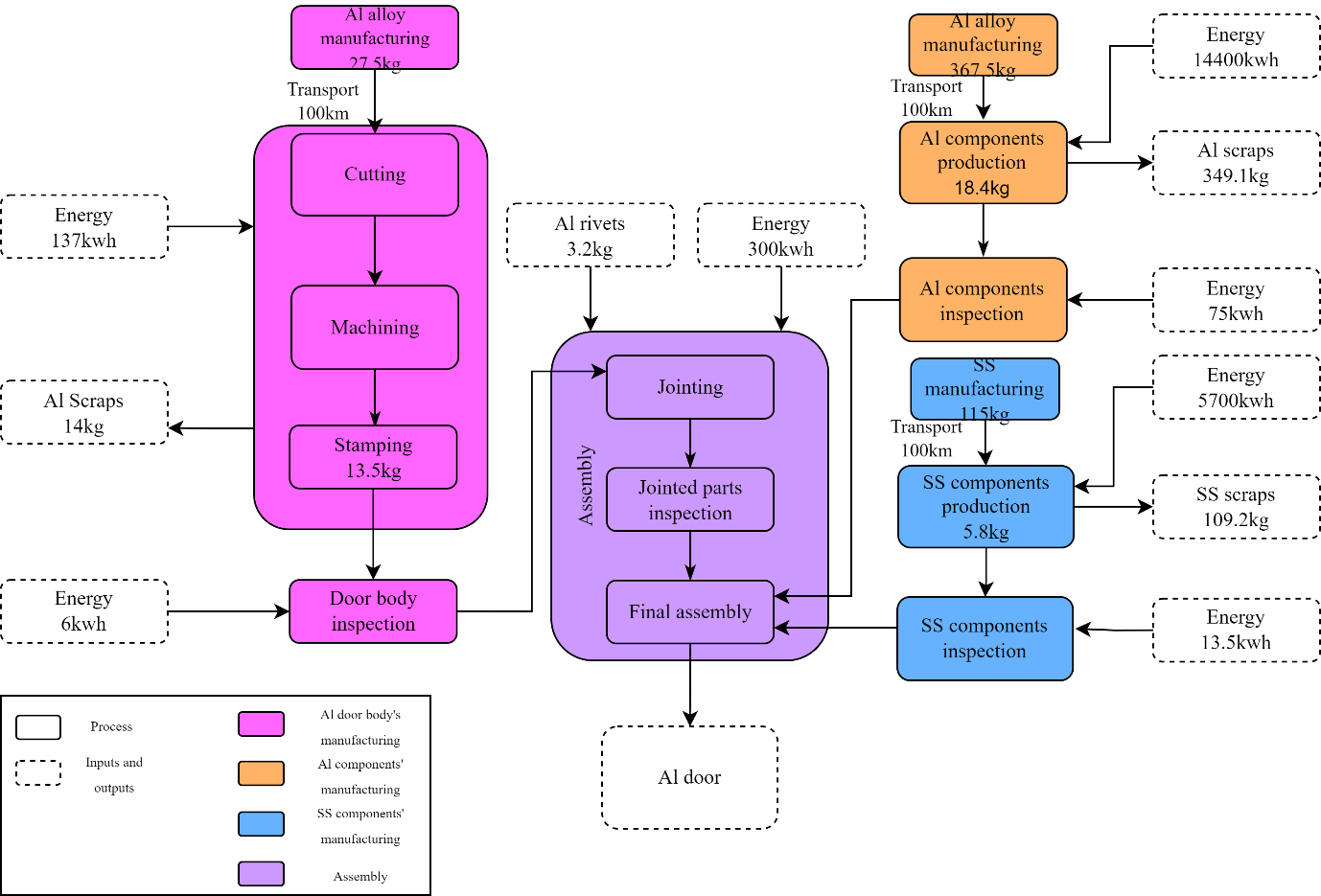


FIGURE ii. The material and energy flows of the aluminium door’s manufacturing. The block colour indicates the type of each production activity: Pink – aluminium door body manufacturing, orange – Al components’ manufacturing, blue – SS components’ manufacturing, purple – Assembly.
